# Bacterial transformation of lignin: key enzymes and high-value products

**DOI:** 10.1186/s13068-023-02447-4

**Published:** 2024-01-03

**Authors:** Jinming Gu, Qing Qiu, Yue Yu, Xuejian Sun, Kejian Tian, Menghan Chang, Yibing Wang, Fenglin Zhang, Hongliang Huo

**Affiliations:** 1 School of Environment, Northeast Normal University, No. 2555 Jingyue Avenue, Changchun, 130117 China; 2 Engineering Lab for Water Pollution Control and Resources Recovery of Jilin Province, Changchun, 130117 China; 3 Engineering Research Center of Low-Carbon Treatment and Green Development of Polluted Water in Northeast China, Ministry of Education, Changchun, 130117 China

**Keywords:** Lignin, Lignin-degrading bacteria, Lignin-degrading enzyme, High-value products

## Abstract

Lignin, a natural organic polymer that is recyclable and inexpensive, serves as one of the most abundant green resources in nature. With the increasing consumption of fossil fuels and the deterioration of the environment, the development and utilization of renewable resources have attracted considerable attention. Therefore, the effective and comprehensive utilization of lignin has become an important global research topic, with the goal of environmental protection and economic development. This review focused on the bacteria and enzymes that can bio-transform lignin, focusing on the main ways that lignin can be utilized to produce high-value chemical products. *Bacillus* has demonstrated the most prominent effect on lignin degradation, with 89% lignin degradation by *Bacillus cereus*. Furthermore, several bacterial enzymes were discussed that can act on lignin, with the main enzymes consisting of dye-decolorizing peroxidases and laccase. Finally, low-molecular-weight lignin compounds were converted into value-added products through specific reaction pathways. These bacteria and enzymes may become potential candidates for efficient lignin degradation in the future, providing a method for lignin high-value conversion. In addition, the bacterial metabolic pathways convert lignin-derived aromatics into intermediates through the “biological funnel”, achieving the biosynthesis of value-added products. The utilization of this “biological funnel” of aromatic compounds may address the heterogeneous issue of the aromatic products obtained via lignin depolymerization. This may also simplify the separation of downstream target products and provide avenues for the commercial application of lignin conversion into high-value products.

## Background

Our excessive dependence on fossil fuels has resulted in climate change and an energy crisis, making the identification and study of renewable and clean energy alternatives an urgent need. Lignocellulosic biomass is the most abundant and renewable source of organic carbon on Earth, with approximately 100 million tons of lignin produced worldwide, corresponding to a total value of about 732.7 million dollars [1]. This makes lignin the best option for achieving sustainable development in the future [2]. In recent decades, research on the conversion of biomass into biofuels and value-added products has shown that lignin can generally be converted into value-added products in two ways. Pathway I involves using lignin as a polymer to produce valuable materials, while pathway II involves depolymerizing lignin into a low-molecular-weight monomer [3] and then subjecting it to various chemical processes, to finally obtain the desired products [4].

Currently, research on lignin has been mainly concentrated in the fields of flame-resistant, sorption, photochemical, electrochemical, and pharmaceutical materials. For example, Song et al. [5] used the Meye method to prepare a thin film composed of graphene nanosheets (GnPs) and multi-walled carbon nanotubes (CNTs) pre-adsorbed with alkali lignin, with the film exhibiting excellent thermal stability and fire resistance. Wang et al. [6] used an adsorbent prepared from modified lignin to realize excellent Pb^2+^ adsorption reaching 130.2 mg/g, which still maintained high adsorption performance after adsorbent recycling. Xing et al. [7] used alkali lignin, pure lignin, and polybutylene adipate terephthalate (PBAT) to produce a UV-protection film that exhibited good UV-shielding performance when it contained 10 wt% lignin. The elemental selenium (Se/LPC) composite electrode prepared by Zhang et al. [8] demonstrated a reversible capacity of 596.4 mAh/g in the second cycle, and the capacity could be maintained at 453.1 mAh/g after 300 cycles, with an average attenuation of 0.08% times/week.

**Fig. 1:**
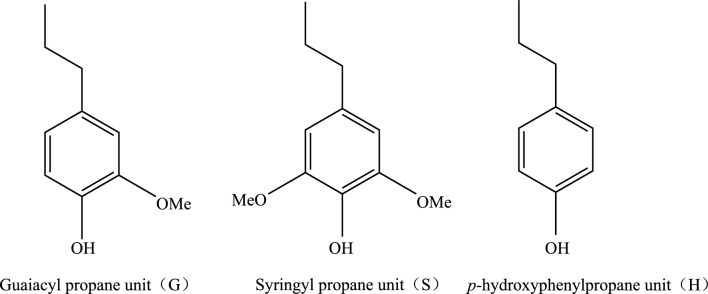
Three basic structural units of lignin

The amount of lignin produced in nature annually is estimated at 0.5–3.6 billion tons [9], though with low availability. Subsequently, effective utilization has become an important issue, due to highly renewable carbon sources in the biosphere. Lignin consists of a complex, amorphous, three-dimensional network of phenylpropane units that are connected by different coupling modes between monomers and oligomers, monomers and monomers, and oligomers and oligomers, with the main connections comprising β-O-4, α-O-4, 4-O-5, β–β, β-5, 5–5 and β-1. Common lignin is mainly composed of three types of phenylpropane structural units, namely, guaiacyl (G), syringyl (S) and *p*-hydroxyphenyl(H) (Fig. 1). The connecting bonds of the three structural units are mainly include aryl ether bonds such as β-O-4 and α-O-4, and carbon–carbon bonds such as β-5 and β–β [10]. Moreover, the structure contains numerous phenolic and aliphatic hydroxyl groups that exist in the free form or are connected with other alkyl or aryl groups via ether bonds [11], endowing the structure with complex amorphous characteristics.

The unique structure of lignin endows it with certain unique properties, such as affecting the free radical reactivity of lignin [12]. The hydrophilic properties of lignin can be enhanced through reactions such as sulfonation and oxidative degradation, which are conducive to the synthesis of anionic surfactants. Lignin exhibits adhesiveness and can be cross-linked and solidified to produce glue. However, the diversity of lignin sources and the complexity of the structure also increase the difficulty of degradation. Therefore, to produce value-added lignin compounds, the depolymerization of lignin remains critical.

The physical and chemical degradation of conventional lignin often requires high-temperature and high-pressure conditions, and it causes formation of inhibitors, such as phenolic compounds, which affect the subsequent enzymatic hydrolysis, resulting in high energy consumption and environmental pollution. The micro-biocatalysis process is usually carried out under mild conditions and can reduce the necessary energy input. This process will be mediated by naturally evolved microbial systems and lignin-degrading enzymes secreted by microorganisms [13], with fungi and bacteria serving as effective microorganisms for degrading lignin. Fungi are major lignin-degrading organisms. At present, studies on lignin degradation by white-rot fungi, brown-rot fungi, and bacteria have been conducted with extensive and in-depth analyses, especially in the fields of phenolic compound pollution remediation, biological pulping, and lignin product refinement. However, a series of problems involved in lignin degradation by fungi have not been solved, including a long pretreatment period, poor adaptability to the environment, and easy contamination by spores. Thus, because bacteria exhibit strong environmental adaptability and biodiversity, their potential for degrading lignin has gradually attracted considerable attention. In studies on the role of bacteria in wood decay, bacteria were found to degrade lignin to a certain extent during the process of degrading lignin to water-soluble polymers. In addition, in the process of lignin degradation process, bacteria can aid fungi in easily degrading lignin and simultaneously removing substances toxic to fungi.

Microbial biodegradation plays a key role in the high-value utilization and transformation of lignin and its derivatives. In this work, the high-value products of lignin were summarized, and we analyzed the lignin-transforming bacteria, lignin-degrading enzymes, lignin high-value products and metabolic pathways. This review may help scholars obtain a better understanding of lignin biotransformation by bacteria and the transformation pathways, as well as provide a theoretical basis for the study of transformation and production of lignin high-value products.

## Lignin-degrading bacteria

At present, among 89 known bacteria, the extent of lignin degradation is known to be between 10 and 89%, as shown in Table 1. Among these, Proteobacteria have the largest number, with 36 strains, followed by Firmicutes with 28 strains and Actinobacteria with 18 strains. Other phyla have fewer strains, such as Acidobacteriota with only 2 strains. Among Proteobacteria, *Escherichia coli*, *Pseudomonas*, *Acinetobacter,* and *Arthrobacter* account for a large proportion of lignin-degrading bacteria, with good lignin-degradation potential. *Enterobacter lignolyticus* SCF1 have demonstrated more than a twofold increase in cell density after lignin addition, with four enzymes involved in upregulated lignin depolymerization, indicating that this strain can depolymerize lignin and use it in growth metabolism [14]. *Pseudomonas putida* mt-2 has also shown good potential for lignin depolymerization, with a good ability to degrade different lignin types. Lignin made from wheat straw, pine, and miscanthus has been modified by fluorescein isothiocyanate [15] by attaching a fluorophore to the lignin polymer. Breakdown of the lignin structure leads to a change in the fluorophore environment, and hence, a change in fluorescence. A strong fluorescence effect was also detected [16], indicating that lignin was degraded. The extent of lignin degradation in corn straw pretreated with an alkali reached 25–30%, and the absorbance increased with time at 280 nm. Among *Acinetobacter*, *Acinetobacter lwoffii* LN4 has demonstrated alkaline-lignin degradation of up to 52% [17], while among *Arthrobacter*, *Arthrobacter* sp. C2 exhibits an alkaline-lignin degradation extent of 65.50% [18], demonstrating the highest degradation extent among lignin-degrading bacteria. Among Firmicutes, *Bacillus* serves as a promising source of new lignin-degrading enzymes, exhibiting the most efficient lignin degradation. *Bacillus cereus* has also shown the highest lignin-degradation efficiency, with a degradation extent of 89% [19]. In addition, *Bacillus* sp. ITRC-S8 can also decolorize sulfate lignin samples, decrease the lignin content, and significantly increase absorbance at 620 nm [20]. In the phylum Actinobacteria, *Streptomyces* sp. S6 was shown to reduce the molecular weight (Mw) of kraft lignin by 55.30% and release aromatic compounds [21]. *Rhodococcus* is also a lignin-degrading genus, with *Rhodococcus jostii* RHA1 serving as a soil bacterium that was initially recognized for degrading polychlorinated biphenyls (PCB). Its lignin-degradation extent in alkali-treated corn stalks was found to range from 25 to 30%, with its absorbance at 280 nm increasing with time, indicating that it exhibited lignin-degrading activity [22].

**Table 1:** Isolated lignin-degrading strains

Phylum	Genus	Bacteria	Lignin type	Concentration/time	Degradation evidence	References
Actinomycetes	*Streptomyces*	*Streptomyces viridosporus* T7A	Corn stalk lignocellulose	5 g/L, 1344 h	Extent of lignin degradation: 36.20%	[16]
			lignin	1.355 g/L, 1344 h	Extent of lignin degradation: 19.70%	
			Wheat straw biomass	–, 504 h	According to ^13^C CP-MAS NMR, the carbohydrate region and the aromatic region have relative fluctuations. Shift resonance between carbon and carboxyl groups, methoxy and carbon in etherification and/or non-etherification	[24]
			Softwood spruce lignocellulose	0.25 g/mL, 2016 h	Extent of lignocellulose degradation: 18.80%; lignin loss 30.90%	[25]
			Hardwood maple lignocellulose	0.25 g/mL, 2016 h	Extent of lignocellulose degradation: 23%; extent of lignin degradation: 19.30%	
			Grass lignocellulose	0.25 g/mL, 2016 h	Extent of lignocellulose degradation: 56.70%; lignin loss of 44.20%	
		*Streptomyces coelicolor* A3(2)	Grass lignocellulose	3.5 g/L, 168 h	To produce the intermediate product APPL	[26]
			Lignin	0.7 g/L, 168 h		
		*Streptomyces* sp. S6	Kraft lignin	–, 144 h	Mw decreased by 55.30%; form aromatic products	[21]
	*Rhodococcus*	*Rhodococcus jostii* RHA1	Fluorescently modified lignin from wheat straw, pine and miscanthus	–, 2 h	Strong fluorescent signal enhancement	[15]
			Lignin made from nitrated wheat straw, pine and miscanthus	–, 2 h	Increased absorbance at 430 nm	
			Sulfate lignin	–, –	Form aromatic dicarboxylic acid products	[27]
			Alkali pretreatment of corn straw lignin	10.5 g/L, 168 h	Extent of lignin degradation: 25–30%; absorbance increased at 280 nm	[22]
		*Rhodococcus jostii* RHA1 VanA-	Straw lignin	–, 120 h	Extent of lignin degradation: 31.60%	[28]
		*Rhodococcus erythropolis*	Lignin made from nitrated wheat straw, pine and miscanthus	–, 20 min	Absorbance increases at 430 nm	[16]
		*Rhodococcus opacus* DSM 1069	Organic solvent lignin	0.3 *w*/*v*%, 168 h	Extent of lignin degradation: 23%	[29]
			Ultrasonic lignin	0.5 *w*/*v*%, 216 h	As the only carbon source for bacterial growth, lignin has loss	
		*Rhodococcus opacus* PD630	Organic solvent lignin	0.3 *w*/*v*%, 168 h	Extent of lignin degradation: 36%	
			Straw lignin	–, 120 h	Extent of lignin degradation: 21.20%; depolymerization forms aromatic hydrocarbons	[28]
		*Rhodococcus jostii* RHA1	Soluble lignin	0.43 g/L, 72 h	Extent of lignin degradation: 18.90%; the β-O-4 bond is broken	[30]
		*Rhodococcus erythropolis* U23A	Alkali pretreatment of lignin	10.5 g/L, 168 h	Extent of lignin degradation: 10–15%; absorbance increased at 280 nm	[22]
	*Arthrobacter*	*Arthrobacter globiformis*	Nitrated wheat lignin	–, 20 min	Increased absorbance at 430 nm	[16]
		*Arthrobacter* sp. C2	Sodium lignosulfonate	–, 164.88 h	Extent of lignin degradation: 40%; the Cα-Cβ bond is broken	[31, 32]
			Alkaline lignin	3 g/L, 216 h	Extent of lignin degradation: 65.50%	[18]
		*Arthrobacter* sp. AXJ-M1	Lignin in black liquor	1.5 g/L, 24 h	Extent of lignin degradation was increased by 30–70%	[32]
	*Pyroactinomyces*	*Thermobifida fusca*	Switchgrass	20 g/L, 100 h	As the only carbon source for bacterial growth	[33]
			Corn stalk	20 g/L, 100 h	As the only carbon source for bacterial growth	
	*Micrococcus*	*Micrococcus yunnanensis* CL32	Alkaline lignin	–, 72 h	Extent of lignin degradation: 39%	[17]
	*Acidomyces pseudoacidus*	*Amycolatopsis* sp. ATCC 39116	Soluble lignin	0.43 g/L, 72 h	Extent of lignin degradation: 24.40%; the β-O-4 bond is broken	[30]
		*Amycolatopsis* sp. 75iv2	Softwood spruce lignocellulose	0.25 g/mL, 2016 h	Extent of lignocellulose degradation: 20.80%; extent of lignin degradation: 34.10%	[16]
			Hardwood maple lignocellulose	0.25 g/mL, 2016 h	Extent of lignocellulose degradation: 32%; extent of lignin degradation: 29.50%	
Proteobacteria	*Enterobacter*	*Enterobacter lignolyticus* SCF1	Sulfate lignin	–, 48 h	Cell density increased by more than 2 times; absorbance change at 280 nm; decreased lignin concentration	[14]
			Alkali pretreatment of corn straw lignin	10.5 g/L, 168 h	Extent of lignin degradation: 17%	[22]
		*Enterobacter soil* sp. nov	Sulfate lignin	0.05%, 48 h	Extent of lignin degradation: 45%	[34]
		*Enterobacter hormaechei* KA3	Corn straw lignin	–, 168 h	Extent of lignin degradation: 32.05%	[35]
		*Enterobacter* BS0669	Corn straw lignin	–, 168 h	Extent of lignin degradation: 10–20%	
		*Enterobacter* BS1957	Corn straw lignin	–, 168 h	Extent of lignin degradation: 20–30%	
	*Pseudomonas*	*Pseudomonas putida* KT2440	Alkali pretreatment of corn straw lignin	10.5 g/L, 168 h	Extent of lignin degradation: 25–30%; absorbance increased at 280 nm	[22]
			Soluble lignin	0.43 g/L, 72 h	Extent of lignin degradation: 23.40%; the β-O-4 bond is broken	[30]
			Alkaline lignin	1.6 g/L, 48 h	Extent of lignin degradation: 30%	[36]
		*Pseudomonas putida* A514	Alkali insoluble lignin	1%(*w*/*v*), –	Grow as the only source of carbon	[37]
		*Pseudomonas putida* NX-1	Sulfate lignin	1 g/L, 168 h	Particle size decreases; absorbance change at 280 nm	[38]
		*Pseudomonas putida* mt-2	Fluorescently modified lignin from wheat straw, pine and miscanthus	–, 2 h	Strong fluorescent signal enhancement	[15]
			Lignin made from nitrated wheat straw, pine and miscanthus	–, 2 h	Increased absorbance at 430 nm	
			Alkali pretreatment of corn straw lignin	10.5 g/L, 168 h	Extent of lignin degradation: 25–30%; absorbance increased at 280 nm	[22]
		*Pseudomonas putida* 33,015	Nitrocellulose lignin	–, 20 min	Increased absorbance at 430 nm	[16]
		*Pseudomonas fluorescens* Pf-5	Alkali pretreatment of corn straw lignin	10.5 g/L, 168 h	Extent of lignin degradation: 15–20%; absorbance increased at 280 nm	[22]
		*Pseudomonas* sp. LD002	Sulfate lignin	0.5 g/L, 72 h	Lignin dye decolorization	[39]
		*Pseudocitrobacter anthropi* MP-4	Alkali lignin	–, 168 h	In the FTIR spectrum, the aromatic framework vibration and C–H deformation vibration, there are aromatic compounds produced. Some characteristic peaks of lignin were found at 1218 cm^−1^. β-O-4 and Cβ disappear	[40]
		*Pseudomonas putida* A514	Alkali lignin	1%, –	Extent of lignin degradation: 27%	[41]
		*Pseudomonas putida* KT3-1	Alkali pretreated lignin (APL)	11.7 g/L, 72 h	*p*-Hydroxybenzoic acid accumulated 20.90% PHA, β-O-4 linkage and small molecule degradation	[42]
		*Pseudomonas putida* B6-2	Alkali pretreated lignin (APL)	11.7 g/L, 72 h	Extent of vanillic acid degradation: 25.40% (72 h); extent of ferulic acid degradation: 100% (48 h); the molar conversion extent of ferulic acid into vanillic acid was 57.20%	
	*Acinetobacter*	*Nocardia autotrophica*	Nitrated pine lignin	–, 20 min	Absorbance increases at 430 nm	[16]
		*Acinetobacter* PC/4	Nitrated wheat lignin	–, 20 min	Absorbance increases at 430 nm	
		*Acinetobacter* sp. ADP1	Alkali pretreatment of lignin	10.5 g/L, 168 h	Extent of lignin degradation: 20–25%; absorbance increased at 280 nm	[22]
		*Acinetobacter john­sonii* LN2	Alkaline lignin	–, 72 h	Extent of lignin degradation: 38.80%	[17]
		*Acinetobacter lwoffii* LN4	Alkaline lignin	–,72 h	Extent of lignin degradation: 52%	
	*Cupriavidus*	*Cupriavidus necator* H16	Alkali pretreatment of corn straw lignin	10.5 g/L, 168 h	Extent of lignin degradation: 15%; absorbance increased at 280 nm	[22]
		*Cupriavidus basilensis* B-8	Sulfate lignin	0.5 g/L, 168 h	Extent of lignin degradation: 38%	[23]
	*Klebsiella*	*Klebsiella pneumoniae* NX-1	Sulfate lignin	1 g/L, 168 h	Particle size decreased and guaiacyl unit decreased. 280 nm absorbance increased; the degradation extent of aromatic compounds was 23.80%	[38]
		*Klebsiella pneumoniae* B-11	Sodium lignosulfonate	1 g/L, 168 h	Extent of lignin degradation: 11.10%	[13]
		*Klebsiella variicola* P1CD1	Sulfate lignin	–, 96 h	Extent of lignin degradation: 30%, aromatic dye decolorization within 24 h;	[43]
	*Ochrobactrum*	*Ochrobactrum tritici* NX-1	Sulfate lignin	1 g/L, 168 h	Particle size decreased and guaiacyl unit decreased. 280 nm absorbance increased; degradation extent of aromatic compounds: 19.40%,	[38]
		*Ochrobactrum pseudintermedium* B-04	Sodium lignosulfonate	1 g/L, 168 h	Extent of lignin degradation: 4.86%	[13]
	*Pandoraea*	*Pandoraea norimbergensis* LD001	Sulfate lignin	0.5 g/L, 72 h	Lignin dye decolorization	[39]
	*Burkholderia*	*Burkholderia* sp*.* H801	Sulfate lignin	1 g/L, 144 h	Extent of lignin degradation: 49.80%	[23]
		*Burkholderia cepacia* B1-2	Alkaline Pretreatment Solution (APL)	11.7 g/L, 48 h	Ferulic acid and vanillic acid are completely consumed	[42]
		*Burkholderia* sp*.* H1	Wheat straw lignin	3.0%(*w*/*v*), 168 h	Extent of lignin degradation: 6.74%	[44]
	*Lanorella*	*Raoultella ornithinolytica* RS-1	Corn straw lignin	–, 168 h	Extent of lignin degradation: 19%; C=O tensile vibration of aromatic ring	[45]
	*Serratia*	*Serratia* sp*.* AXJ- M	Lignin in black liquor	1.5 g/L, 24 h	Extent of lignin degradation: 30%	[32]
	*Stenotrophomonas*	*Stenotrophomonas* sp*.* S2	Alkaline lignin	–, 72 h	Extent of lignin degradation: 50%	[46]
Firmicutes	*Bacillus*	*Bacillus* sp*.* LD003	Sulfate lignin	0.5 g/L, 72 h	Lignin dye decolorization	[39]
		*Bacillus pumilus* C6	Sulfate lignin	–, 168 h	Mw decreased; GGE extent of lignin degradation: 35.10%	[47]
		*Bacillus atrophaeus* B7	Sulfate lignin	–, 168 h	Mw decreased; the degradation extent of GGE was 27.50%	
		*Bacillus* sp*.* ITRC-S8	Sulfate lignin	0.5 g/L, 144 h	Sample decolorization; Increased absorbance at 620 nm	[20]
		*Bacillus ligninphilus*	Alkaline lignin	1 g/L, 168 h	Extent of lignin degradation: 38.90%; extent of lignin degradation: 30%; form monomer aromatic compounds	[38]
		*Bacillus subtilis*	Alkali pretreatment of corn straw lignin	10.5 g/L, 168 h	Extent of lignin degradation: 5–10%; absorbance increased at 280 nm	[22]
		*Bacillus megaterium*	Alkali pretreatment of corn straw lignin	10.5 g/L, 168 h	Extent of lignin degradation: 5–10%; absorbance increased at 280 nm	
		*Bacillus altitudinis* SL7	Alkaline lignin	3 g/L, 120 h	Extent of lignin degradation: 44%; lignin decolorization; alcohol-OH bond stretching of phenol	[48]
		*Bacillus flexus*	Sulfate lignin	0.4 g/L, 216 h	Extent of lignin degradation: 20%	[23]
		*Bacillus aryabhattai* BY5	Alkaline lignin	–, 72 h	Extent of lignin degradation: 53.50%	[17]
		*Bacillus flexus* RMWW II	Alkali lignin	0.1 g/L, 216 h	Extent of lignin degradation: 97.10%	[49]
		*Bacillus subtilis* ACCC 11089	Alkali lignin	0.5 g/L, 360 h	Extent of lignin degradation: 17.30%; form aromatic compounds; the C4 ether and the Cα-Cβ bond are broken	[50]
			Hydroxylated lignin	0.5 g/L, 360 h	Extent of lignin degradation: 11.40%; form aromatic compounds; the C4 ether and the Cα-Cβ bond are broken	
			BOC lignin	0.5 g/L, 360 h	Extent of lignin degradation: 24.60%; form aromatic compounds; the C4 ether and the Cα-Cβ bond are broken	
		*Bacillus amyloliquefaciens* SL-7	Lignin in tobacco straw	3 g/L, 360 h	Extent of lignin degradation: 28.55%; total carbon content goes down	[51]
		*Bacillus sonorensis* B-45	Sodium lignosulfonate	1 g/L, 168 h	Extent of lignin degradation: 7.68%	[13]
		*Bacillus* sp*.* strain BL5	Alkali lignin	0.4%, 12 h	Extent of lignin degradation: 27.04%; –OH bond stretching between alcohol and phenol; form ethers, phenols and alcohols	[52]
		*Bacillus cereus* AH7-7	Sulfate lignin	1 g/L, 144 h	Extent of lignin degradation: 25.90%	[53]
		*Bacillus aryabhattai*	Kraft lignin	0.5 g/L, 336 h	The degradation extent was 84%. Absorbance changes at 280 nm	[54]
		*Bacillus cereus*	Kraft lignin	1 g/L, 72 h	Extent of lignin degradation: 89%; decolorization extent: 40%	[19]
		*Bacillus subtilis* TR-03	Alkali lignin	2 g/L, 36 h	Extent of lignin degradation: 26.72%; decolorization extent: 71.23%; 280 nm absorbance increased; aromatic skeleton spectrum band vibration	[55]
		*Bacillus cereus* TR-25	Alkali lignin	2 g/L, 36 h	Extent of lignin degradation: 23%; decolorization extent: > 50%; 280 nm absorbance increased; aromatic skeleton spectrum band vibration	
		*Bacillus subtilis* S11Y	Alkaline lignin	–, 72 h	Extent of lignin degradation: 20%	[46]
		*Bacillus velezensis* TSB1	Kraft lignin	0.6 g/L, 144 h	Extent of lignin degradation: 40.39%; extent of lignin degradation: 56.16%	[56]
	*Bacillus spinoides*	*Paenibacillus glucanolyticus* SLM1	Bioselective lignin	0.2%, 400 h	Mw decreased; weight reduction	[57]
		*Paenibacillus glucanolyticus* 5162	Bioselective lignin	0.2%, 400 h	Mw decreased; weight reduction	
		*Paenibacillus* sp*.*	Alkali pretreatment solution	10.5 g/L, 168 h	Extent of lignin degradation: 12%	[22]
	*Clostridium*	*Clostridium thermocellum* ATCC 27405	Populus lignin	–, –	The amount of β-O-4 chain reaction decreased; the S/G index increased	[58]
	*Bacillus geodesis*	*Geobacillus thermodenitrificans* Y7	Switchgrass lignin	7.0 g/L, 120 h	Extent of lignin degradation: 17.21%	[44]
Acidobacter	*Citrobacter*	*Citrobacter freundii*	Alkali pretreatment of corn straw lignin	10.5 g/L, 168 h	The degradation extent is 5–10%; absorbance increased at 280 nm	[22]
	*Thermophilic anaerobes*	*Caldicellulosiruptor bescii*	Switchgrass	0.5%, 240 h	Form monomer aromatic compounds	[59]
	*Azotobacter*	*Azotobacter vinelandii* NRS 16	Alkali pretreatment of lignin	10.5 g/L, 168 h	Extent of lignin degradation: 5–10%	[22]
		Microbial Alliance LDC	Rice straw lignin	–, 168 h	Extent of lignin degradation: 31.18%	[60]
		AC-1	Straw lignin	–, 360 h	Extent of lignin degradation: 20.12%	[61]

In addition, the bacteria can degrade lignin: *Paenibacillus, Actinomyces, Clostridium, Caldicellulosiruptor*, *Cupriavidus*, *Azotobacter, Citrobacter, Klebsiella, Ochrobactrum*, *Pandoraea*, *Burkholderia*, *Micrococcus, Raoultella*, *Actinomyces, Serratia, Geobacillus*, *Stenotrophomonas*, and some common mixed bacterial systems. Among these, the extent of sulfate-lignin degradation by *Cupriavidus basilensis* B-8 was 38%, and that of *Burkholderia* sp. H801 was 49.80% [23]. The lignin degradation extent of *Raoultella ornithinolytica* MP-132 was shown to be 53.20%. Thus, lignin-degrading bacteria are widely distributed in the natural environment, and the types of bacteria are diverse. Bacterial mixed systems also exhibit a lignin-degrading ability, indicating the potential for diverse microbial lignin degradation methods.

Genomics plays an increasingly important role in the study of bacterial transformation of organic matter, providing more rational assessment and prediction of bacterial biotransformation ability and substrate preference. Due to the substantial differences in lignin conversion ability of the abovementioned bacteria, and to further explore and clarify the specific lignin conversion functions of the bacteria, the whole genome sequencing of these bacteria was summarized in this review. The genome characteristics of some lignin-degrading bacteria are listed in Table 2.

**Table 2:** Genome characteristics of lignin-degrading bacteria

Bacteria	Total sequence Length (bp)	GC content (%)	N50 (bp)	CDS	Gene number (gene data base)	References
*Agrobacterium* sp*. strain* S2	9,722,071	58.90	5151	–	–	[62]
*Amycolatopsis* sp*.* ATCC 39116	8,442,518	71.90	–	8264	–	[63]
*Streptomyces viridosporus* T7A ATCC 39115	8,278,598	72.50	–	7552	–	[64]
*Paenibacillus* sp*.*	7,187,707	43.50	353,719	–	–	[65]
*Streptomyces* sp*.* strain S6	6,420,514	71.23	2057	9405	3822 (COG)	[21]
*Klebsiella* sp*.* strain BRL6-2	5,801,355	55.24	–	5296	4599 (COG) 4904 (Pfam)	[66]
*Mangrovibacterium lignilyticum* sp. nov	5,634,442	43.40	795,598	–	–	[67]
*Klebsiella variicola* P1CD1	5,633,647	57.30	–	5451	–	[68]
*Raoultella ornithinolytica* strain S12	5,522,044	57.47	–	4875	3978 (COG)	[43]
*Bacillus cereus* AH7-7	5,328,700	35.36	–	–	–	[53]
*Ochrobactrum* sp*.*	5,310,057	56.20	150,153	–	–	[65]
*Pseudomonas* sp*.* Hu109A	5,131,917	62.60	–	4689	4097 (COG) 3577 (GO) 2681(KEGG)	[69]
*Enterobacter lignolyticus* SCF1	4,814,049	57.02	–	–	3743 (COG)	[14]
*Arthrobacter* sp*.* C2	4,555,954	66.23	–	–	–	[31]
*Arthrobacter* sp*.* strain RT-1	4,545,853	65.69	282,801	–	4068 (KEGG)	[70]
*Acinetobacter calcoaceticus* CA16	4,110,074	38.69	–	3798	–	[71]
*Burkholderia* sp*.* Strain LIG30	4,002,050	66.40	84,253	4996	–	[72]
*Meridianimaribacter* sp*.* CL38	3,332,696	33.13	595,671	2930	2230 (COG) 1313 (KEGG)	[73]
*Pandoraea* sp*.* ISTKB	2,139,250	62.05	132,761	5356	1351 (KEGG) 4603 (Pfam)	[74]

As a lignin-degrading bacterium, *Pseudomonas* has exhibits catabolic potential to break down a variety of natural aromatic compounds, destroying guaiacyl-propane lignin units and decomposing benzene rings [75]. Among these, *Pseudomonas* sp. Hu109A contains a chromosome ring with a genome size of 5.13 Mb and a GC content of 62.60%. The genome contains 4789 genes, of which 4698 genes (accounting for 88.90% of the genome) are considered as protein-coding sequences (CDS), 73 are annotated as tRNA genes, and 18 are annotated as rRNA genes. In addition, through gene function annotation, 4097 genes (87.20%) were annotated to the COG database, 3577 genes (76.13%) have been annotated to the GO database, and 2681 genes (57.06%) were annotated to the KEGG database [69]. According to genomic annotations and related references, 59 structural genes encoding lignin-degrading enzymes and 53 enzymes involved in PHA synthesis were identified. Genes related to lignin degradation include dioxygenase, *O*-methyltransferase, peroxidase, hydroxylase, decarboxylase, oxidase, reductase, catalase, isomerase and dehydrogenase. In addition, four gene clusters have been identified in the genome of strain Hu109A, two of which were found to be involved in lignin degradation. The strain showed higher PHA yields when grown on lignin as the only carbon source. When 1 g/L lignin was used as the sole carbon source, the maximum observed PHA production value was 103.68 mg/L, which increased to 186 mg/L with an increase in lignin concentration to 3 g/L, revealing a higher efficiency of the lignin biotransformation pathway.

*Streptomyces* sp. strain S6 has a genome size of 6,420,514 bp, CDS of 9,405, 6,064 predictive functional proteins, and 3,341 hypothetical proteins. According to the COG functional categories, general processes and metabolic pathways can be primarily related to amino acids, carbohydrates, fatty acids and lipids dominate. Secondary metabolite biosynthesis, transport, and catabolism were found to be consistently common among the subsystem characteristics, indicating that strain S6 had the ability to metabolize lignin or aromatic compounds. The presence of catalase-peroxidase and laccase in the strain S6 genome could enable the synergistic degradation of lignin, reducing the lignin weight by 55.30% and generating aromatic compounds [21]. In addition, *Streptomyces viridosporus* T7A ATCC 39115 was found to contain a total genome size of 8,278,598 bp and GC content of 72.50%, with 7552 candidate protein-coding genes identified. The genome contains many genes that code for enzyme homologues that can deconstruct plant biomass. The COG notes showed that 8.35% of the predicted proteins were involved in carbohydrate transport and metabolism. Genes encoding putative lignin-degrading enzymes, such as heme peroxidase, dye-decolorizing peroxidases (DyPs) have been identified [64] and are involved in the degradation of grass lignocellulose, with a degradation extent of 44.20% [25].

In addition, the genome size of other lignin-degrading strains was approximately 5 Mb. The maximum size of the genome of *Agrobacterium* sp. strain S2 was found to be 9.70 Mb, and the minimum size of *Pandoraea* sp. ISTKB was only 2.10 Mb. Furthermore, *Bacillus cereus* AH7-7, *Acinetobacter calcoaceticus* CA16, and *Meridianimaribacter* sp. CL38 demonstrated relatively low GC contents, in the range of 30 to 40%.

In summary, the function of bacteria to transform lignin can be related to the genes encoding lignin peroxidase and other enzymes responsible for lignin degradation. These provide genomic material for the in-depth analysis of the lignin biotransformation mechanism at the gene level. However, the whole genome sequencing of lignin-degrading bacteria has not been carried out, and further research on whole genome sequencing is needed.

## Lignin-degrading enzyme

Enzymatic reactions play an important role in the process of bacterial biotransformation process of organic matter. As early as 1930, Phillips et al. [76] reported on the decomposition of lignin by “soil microorganisms”, which were possibly bacteria. However, the functions and effects of lignin-degrading enzymes have only been identified in recent decades. In general, bacteria growing in lignin will secrete various oxidases to assist in the depolymerization or modification of lignin, among which DyPs and laccase play an important role in bacterial depolymerization of lignin. This review summarizes in detail the enzymes that play a role in the process of lignin conversion by bacteria, as shown in Tables 3 and 4.

**Table 3:** Dye-decolorizing peroxidase for lignin degradation

Category	Protease	Substrate	Degradation evidence	Entry number	References
Subfamily A	BsDyP	ABTS^a^	53.50% of VGE is converted to veratral	WP_003222196	[81]
		VGE			
	TfuDyP	ABTS	The β-aryl ether monomer is produced at m/z 343.2	–	[83]
		GGE^a,b^			
		Kraft lignin			
	SviDyP	2,6-DMP^b^	Triarylmethane dyes, anthraquinones and azo dyes can be degraded under neutral to alkaline conditions	KF444221	[84]
Subfamily B	DypB	ABTS^a,c^	The DyPB gene deletion mutant significantly reduced lignin degradation and produced a small amount of vanillin	–	[85]
		GGE			
		β-aryl ether lignin dimers^a,c^			
		Kraft lignin			
		Alkali lignin^a^	Extent of lignin degradation: 27%	–	[41]
	DyP1B	Guaiacol^a,b^	Low molecular weight aromatic products are released	Q4KAC6_PSEF5	[86]
		Kraft lignin			
		Straw cellulose			
	PpDyp	Syringaldehyde^a^	The catalytic efficiency of phenolic compounds was enhanced and the yield was increased	–	[90]
		GGE DMP			
		Kraft lignin			
Subfamily C	DyP2	ABTS^a,b^	Ability of DyP2 to degrade lignin model dimers containing β-O-4 junctions	–	[91]
		GGE			

^a^Gene recombination

^b^Enzyme purification

^c^Gene knockout

**Table 4:** The laccase and other enzymes for lignin degradation

Category	Protease	Substrate	Degradation evidence	Entry number	References
Laccase	Lac	ABTS^a,b^	Acid precipitable polymerized lignin, production of vanillin	–	[26]
		GGE			
		VGE			
		Ethanosolv lignin			
	Lac4	Guaiaco^a,b^	Extent of lignin degradation: 38%; a large number of low molecular weight aromatic compounds were detected	WP_028724718.1	[96]
		Lignin			
	SilA	Sinapic acid	Polymerization of lignin and lignans	–	[95]
		Kraft lignin			
Multi-copper oxidase (Laccase-like multicopper oxidase, LMCO)	CueO	ABTS^a,b^	The production of vanillic acid	6EVG	[65]
		GGE			
		Ca-lignosulfonate			
	CotA	Sinapic acid^a^	–	NP_388511.1	[113]
	Pp-CopA	Guaiacol^b^	The new peak of electrospray mass spectrometry contains vanillic acid	Q88C03	[100]
	Pf-CopA	GGE			
		Ca-lignosulfonate			
Lignin peroxidase	LiP	2,4-DCP	Acid precipitation polymerized lignin is produced, and 3,4-dihydroxyphenylalanine is oxidized	–	[114]
	ALiP-P3	1,2-Diaryl propane	Break the Cα-Cβ bond of the β-O-4 model compound	–	[109]
		Corn lignin^b^			
	LiP	Veratryl alcohol	Veratryl alcohol was oxidized to veratraldehyde at 310 nm; strong absorption at A_280_ nm leads to degradation of aromatic compounds	MF093751	[38]
		Kraft lignin			
β-aryletherase	LigD	GGE^a^	Generates alpha-(2-methoxyphenoxy)-beta-hydroxypropiovanillone (MPHPV)	–	[115]
	LigE	MPHPV^a^	Production of guaiacol and α-glutathione beta-hydroxyprothiolone (GS-HPV)	–	[116]
	LigF				
Bacterial dioxygenase	LigAB	3-o-Methylgallate	Formation of 4-carboxyl-2-hydroxy-6-methoxy-6-oxyhexa-2, 4-dioleate (CHMOD) and 2-pyranone-4, 6-dicarboxylate (PDC)	–	[105]
	LigZ	DDVA	To form 5-carboxylvanillic acid and 4-carboxyl-hydroxypentaenoic acid	–	[107]
	DesB	3-o-Methylgallic acid	The production of 4-oxalomesaconate	AB190989	[117]
	DesZ	3-o-Methylgallic acid	The production of PDC	–	[117]
	pcaHG	PCA	The production of β-carboxymuconate	–	[118]
	C23O	Catechol	The production of 2-hydroxymuconic acid semialdehyde	–	[119]
	BphC	Alkylated 2, 3-dihydroxybiphenyls (DHB)	The production of 6-phenyl HODA	–	[106]
	AphC	Catechol	Meta-lysis products are generated	3LM4	[106]
	LsdA	Lignostilbene^b^	The intermediate lignostilbene was converted into vanillin	–	[120]
	PcaA	PCA	The reaction products showed ketoenol tautomerism	–	[121]
Catalase-peroxidases	Amyco1	Miscanthus giganteus lignin	Phenolic lignin model compounds and produce acid-precipitated polymer lignin	–	[110]
Monocopper polyphenol oxidase	Tfu1114	2,6-DMP	Absorption is increased at A_280nm_ and aromatic substances are present	–	[111]
		Alkaline lignin			
		Sugarcane bagasse			
Manganese-dependent superoxide dismutase	MnSOD	Organosolv^a^	The resulting product is formed by oxidative cleavage and o-demethylation of aryl-cα and Cα-Cβ bonds	2RCV	[112]
		Kraft lignin			
LPMO	LPMO-AOAA17	Guaiacol	The ultraviolet spectrum of guaiacol oxidation at 470 nm showed that brown oxidation products were formed. Formation of 3, 4-dimethoxybenzaldehyde (veratraldehyde)	–	[122]
		2,6-DMP			
		3,4-dimethoxybenzyl alcohol			
		Vanillyl alcohol			
		GGE			

^a^Gene recombination

^b^ Enzyme purification

### Dye-decolorizing peroxidases

DyPs are newly discovered members of the heme-containing peroxidase family and have a strong lignin-degrading ability. At present, there are two methods to classify DyP peroxidase, one is phylogenetic analysis based on amino acid sequence similarity, and the other is structure-based sequence alignments [77]. The former classifies DyP peroxidase into class A, B, C and D [78], among which class A–C DyPs mainly exist in bacteria, while class D DyPs mainly exist in fungi. Class A DyPs contain twin arginine translocation (Tat) signal sequences in the N terminus, while Class B and C DyPs do not contain typical secreted signal peptides and are classified as intracellular proteins [79]. However, in the phylogenetic tree, Class C DyPs is far from Class A and B DyPs. There are few reports about class C DyPs, which makes it difficult to define the characteristics of class C DyPs.

Tat of DyP type A peroxidase is a protein secretion transmembrane transport system that contains a highly conserved double arginine motif signal peptide in the secreted protein signal peptide, indicating that DyPs can be secreted into the extracellular environment and participate in extracellular lignin depolymerization [80]. For example, a DyP (BsDyP, entry number: WP_003222196) found in *Bacillus subtilis* KCTC2023 has demonstrated oxidative activity toward 2,2′-azino-bis(3-ethylbenzothiazoline-6-sulfonic acid (ABTS) and reactive blue dyes. Furthermore, BsDyP has shown an efficient ability for the decomposition of veratryl glycerol-β-guaiacyl ether (VGE), and a dimerized lignin model compound containing β-O-4 bond, converting 53.50% of VGE into veratrol aldehyde within 2 h [81]. BsDyP decomposes VGE by cracking the Cα–Cβ bond, and the aliphatic hydroxyl group on the benzene ring is oxidized to aldehyde hydroxyl group, resulting in veratraldehyde. TfuDyP from *Thermobifida fusca*, a class A DyP, has been found to be active against sulfate lignin, guaiacylglycerol-β-guaiacyl ether (GGE), guaiacol, and 2,6-dimethoxyphenol, forming new products [82]. TfuDyP transforms guaiacylglycerol-β-guaiacyl ether into β-aryl ether monomer, and C–C coupling with phenolic hydroxyl benzene ring forms biphenyl structure, which is an oxidative dimerization reaction [83]. In addition, *Saccharomonospora viridis* DSM 43017 also contains class A DyP peroxidase (SviDyP, entry number: KF444221), which can degrade triarylmethane, anthraquinone, and azo dyes under neutral to alkaline conditions, with activity at pH values of 5 to 10 and temperatures of 50 ℃ to 80 ℃. It was proved by cyclic voltammetry that the azo bond of the dye was broken to form amines, and then oxidation reaction was carried out [84].

Class B DyPs carry out redox reactions by attacking the Cα–Cβ bond of phenolic lignin compounds, attracting considerable attention due to their multifold functionality and high activity, with class B DyP serving as a representative DyPB. Ahmad et al. [85] found that the DyPB-deficient *R. jostii* RHA1 mutant significantly reduced lignin degradation, indicating that DyPB played a key role in lignin decomposition. During the reaction, the aliphatic hydroxyl group on the benzene ring of β-aryl ether changed into carbonyl group, indicating that the C(α)–C(β) bond was cracked and a small amount of vanillin was produced. Lin et al. [41] identified two DyPB enzymes from *P. putida* A514, which did not contain typical signal peptides, and had lignin-depolymerization activity independent of Mn^2+^. The high synergistic activity of DyPB and A514 was found to promote cell growth and lignin degradation. DyPB decomposes lignin into low molecular weight lignin-derived compounds, which leads to the cleavage of aryl ether bonds such as β-O-4 and α-O-4, and C–C bonds such as β-5 and β–β. *Pseudomonas fluorescens* Pf-5 was found to contain two types of DyPB [86], and the oxidation activities of DyP1B (entry number: Q4KAC6_PSEF5) against Mn(II) and sulfate lignin were observed, which cracked lignocellulose and generated β-aryl ether lignin dimer containing G unit and H unit. PpDyP, another important B-type DyP, namely, PpDyP was also found in *P. putida* MET94 [87], which can transform some phenolic compounds related to lignin, such as GGE, mainly by mediating the one-electron oxidation of phenols, leading to the formation of free radical intermediates or quinones, and then coupling with other substrate molecules to generate new substances. In addition, it was discovered that the 6E10 variant containing PpDyP improved the decomposition ability of 2,6-dimethoxyphenol by 100 times.

There are currently 24 proteins have been annotated in PeroxiBase as Class C, with several known class C DyPs consisting of Proteobacteria, Actinobacteria, and Cyanobacteria [88]. For example, DyP2, a strong peroxidase found in *Amycolatopsis* sp*.* 75iv2, serves as a typical representative of Class C DyPs [89]. DyP2 has demonstrated high manganese peroxidase activity and lignin depolymerization ability, with broad spectrum of substrate specificity, demonstrating peroxidase activity toward phenol, azo dyes and anthraquinone dyes.

### Laccase

Laccase is a multicopper oxidase that is widely used in the biological industry, including in bioreactors, lignin modification, biobleached pulp, and biofuel cells [92]. Laccase coding genes can be widely found in bacteria, especially *Streptomyces*, *Bacillus*, and *Pseudomonas* [93], and contain four typical copper-binding sites. In the presence of molecular oxygen, the lignin substrate will lose and an electron to generate phenoxy free radicals, resulting in side-chain breakage of lignin phenolic structural units, including Cα–Cβ bond breakage and Cα oxidation. Furthermore, bacterial laccase has demonstrated higher heat resistance than fungal laccase, and making it suitable for industrial applications as a catalyst [94].

In the study of lignin degradation by laccase, the ability of laccase to depolymerize lignin has been directly quantified by characterizing lignin or lignin-related compounds, with its potential as a lignin-modifying enzyme can be indirectly assessed by characterizing oxidation indicators such as ABTS and various dyes. Majumdar et al. [26] reported on four laccases from *Streptomyces coelicolor* A3(2), *Streptomyces lividans* TK24, *Streptomyces viridosporus* T7A and *Amycolatopsis* sp. 75iv2, which could degrade a phenolic model compound (LM-OH) into intermediates such as vanillin, which is caused by the free radical reaction initiated by SLac, and the Cα-Cβ bond of downstream compounds is broken. The four laccases demonstrated the highest activity at pH of 8.0, and could also degrade a phenolic model compound (LM-OH) to intermediate products such as vanillin. Moya et al. [95] discovered a bacterial laccase SilA in *Streptomyces ipomoea* CECT 3341. Lignin treated with SilA and lignans polymerized under alkaline conditions, which showed that SilA could modify industrial waste lignin into value-added polymer under alkaline conditions. In addition, laccase Lac4 was found in the *Pantoea ananatis* Sd-1 genome (entry number WP_028724718.1). After the addition of ABTS, 38% of lignin was degraded by Lac4, and GC–MS analysis showed that low-molecular-weight aromatic compounds, including 1,4-phenyldicarboxylic acid, phenylpropionic acid, and phenol were generated [96].

Laccase-like multicopper oxidase (LMCO) can catalyze the transfer of four electrons from each substrate molecule to form oxidation products by reduction-splitting dioxygen bonds [97]. In this catalytic cycle, one oxygen molecule will be reduced to two water molecules and four substrate molecules will be oxidized to form four corresponding reactive free-radical intermediates; thus, copper in the catalytic core of laccase plays an important role. CueO (entry number: 6EVG), a multicopper oxidase in *Ochrobactrum* sp. shows activity for oxidation of 2,2′-dihydroxy-3,3′-dimethoxy-5,5′-dicarboxybiphenyl (DDVA), and decarboxylation occurs during this process to generate oxidized dimeric products. In addition, CueO also shows activity for oxidation of Ca-lignosulfonate to generate vanillic acid, which resulted in the side-chain breakage of lignin phenolic structural units, including Cα–Cβ bond breakage and Cα oxidation [65]. CotA (entry number: NP_388511.1), another well-studied LMCO, was initially thought to be a protein related to the spore envelope, but it was later determined to exhibit structural characteristics similar to those of multicopper oxidase [98]. CopA, a part of the copper resistance operon in *Pseudomonas syringae* pv. *Tomato* and has also been identified as a potential lignin oxidase [99]. Granja et al. [100] discovered that CopA (entry number: Q88C03) in *P. putida* KT2440 and *Pseudomonas fluorescens* PF-5 could oxidize lignin model compound GGE and 5,5'-dehydrostilbenate (DDVA) to dimers, converting lignosulfonate into vanillic acid, however, the ability of a *P. putida* KT2440 mutant with the deletion of CopA encoding gene to generate aromatic compounds was reduced. This confirmed the involvement of CopA in lignin oxidation.

### β-Aryl etherase

Β-Aryl ether (e.g., GGE) is a dimer compound that contains the β-O-4 bond, which dominates the lignin structure, with a content of about 50–70% content [101]. Thus, β-aryl ether plays an important role in lignin degradation. β-aryl etherase (also known as glutathione S-transferase, GST) systems such as LigD (Cα-dehydrogenase), LigF (β-etherase), LigG (glutathione lyase) and LigE/P (glutathione reductase) can cleave β-aryl ether [102]. This system was first discovered in *Sphingobium* sp. SYK-6 [103]. LigD and LigEFG were found to decompose β-aryl ether into vanillic acid by cracking β-O-4, and LigEFG was further involved in the reduction of glutathione [104]. In addition, LigF-active enzymes were found in *Novosphingobium* sp. PP1Y, *Novosphingobium aromaticivorans* DSM12444, and *Sphingobium* sp. SYK-6. These enzymes can catalyze the cracking of four dimers, namely, guaiacyl-β-guaiacyl, guaiacyl-β-syringyl, syringyl-β-guaiacyl, and syringyl-β-syringyl [104]. These enzymes usually act after the conversion of lignin into low-molecular-weight lignin oligomers, which can be further converted into usable biochemicals.

### Bacterial dioxygenases

Dioxygenase plays an important role in the lignin transformation process, and is involved in the decomposition of PCA, catechol, and gallic acid (GA) through an aromatic ring. *Sphingomonas paucimobilis* SYK-6 cracked the benzene ring of MGA through DesZ and LigAB, and the hydroxyl group was oxidized to carbonyl group, resulting in 4-carboxyl-2-hydroxy-6-methoxy-6-oxyhexa-2, 4-dioleate (CHMOD) [105]. In *Pseudomonas* sp.CF600, the meta-cleavage pathway of catechol was found, and catechol was transformed by catechol 2,3 dioxygenase (C23O) to produce 2-hydroxyl mucosal hemialdehyde [106]. In addition, a study found that in *S. paucimobilis* SYK-6, with a biphenyl compound 2,2′-dihydroxy-3,3′-dimethoxy-5,5′-dicarboxylic biphenyl (DDVA) as the substrate, four enzymes with different functions were involved in the initial steps to degrade biphenyls. Among these, diol exodioxygenase (LigZ), as a conversion tool, catalyzes the break of C–C bond adjacent to the hydroxyl group, thus cleaving the upstream products to produce 5-carboxylvanillic acid and 4-carboxyl-hydroxypentaenoic acid, and the 5-carboxylvanillic acid was converted into vanillic acid by other enzymes [107]. These findings suggested that dioxygenase can assist bacteria in lignin degradation.

### Other bacterial enzymes involved in lignin degradation

Lignin peroxidase (LiP) serves as the main lignin-degrading enzyme in lignin biodegradation. LiP is a heme protein, which is an enzyme that catalyzes the oxidation of substrate with H_2_O_2_ as electron acceptor. LiPs are activated by H_2_O_2_ to attack lignin through intermediate radicals [108]. However, most studies found LiPs to be widespread in fungi, with a small number found in bacteria. For example, highly active LiP (entry number: MF093751) was detected in *Ochrobactrum tritici* NX-1, which was found to degrade 19.4% of the sulfate lignin, with increased absorbance at A280 nm [38]. Moreover, in *S. viridosporus* T7A ATCC 39115, a lignin peroxidase, ALiP-P3, was found to break the Cα–Cβ bond of the β-O-4 model compounds during the degradation of lignin. Its cleavage mode was found to be similar to that of *Thermomonospora* sp lignin peroxidase [109]. Manganese peroxidase (MnP) is a class of heme-containing peroxidase with a catalytic cycle that uses Mn^2+^ as an electron donor to convert Mn^3+^. Mn^3+^ will diffuse from the enzyme surface to oxidize the phenolic substrate. Xu et al. detected highly active MnP when studying the degradation of lignin by *P. putida* NX-1. However, because the NX-1 strain secreted laccase and lignin peroxidase at the same time, the degradation of lignin was not found to be caused by MnP. Other studies also detected the activity of MnP in several bacteria [22], but no relevant data showed that MnP was involved in lignin depolymerization.

Brown et al. [110] found that *Amycolatopsis* sp. 75iv2 secreted a heme-containing enzyme known as catalase-peroxidase (Amyco1), transforming phenolic lignin model compounds when incubated with cellulosic materials. We also confirmed that this enzyme acts on lignin compounds. However, it remains unclear whether bacteria often use catalase-peroxidase to degrade lignin. In addition to the typical lignin depolymerase mentioned above, different types of oxidases were found in *T. fusca* and *Sphingobacterium* sp. T2, exhibiting activity similar to that of laccase for lignin modification. Chen et al. [111] identified single copper polyphenol oxidase (Tfu1114) in *T. fusca*, which could reduce the total phenol content of alkali lignin. LC–MS was used to detect the changes in the lignin structure of bagasse, indicating the production of eugenol and pseudo-aromatic alcohol. This process may be that Tfu1114 destroyed the C–C and C–O bonds adjacent to aromatic rings. Furthermore, for bacterial manganese-dependent superoxide dismutase (MnSOD, entry number:2RCV) in *Sphingobacterium* sp. T2, both MnSOD1 and MnSOD2 were found to exhibit activity toward sulfate lignin and lignin model compounds through proteomic analysis, and the oxidation products produced by oxidative cleavage and *o*-demethylation of aryl–Cα and Cα–Cβ bonds were detected [112]. However, whether MnSOD1 and MnSOD2 can serve as typical lignin depolymerase still needs to be determined.

## Lignin high-value products and their formation pathway

**Fig. 2:**
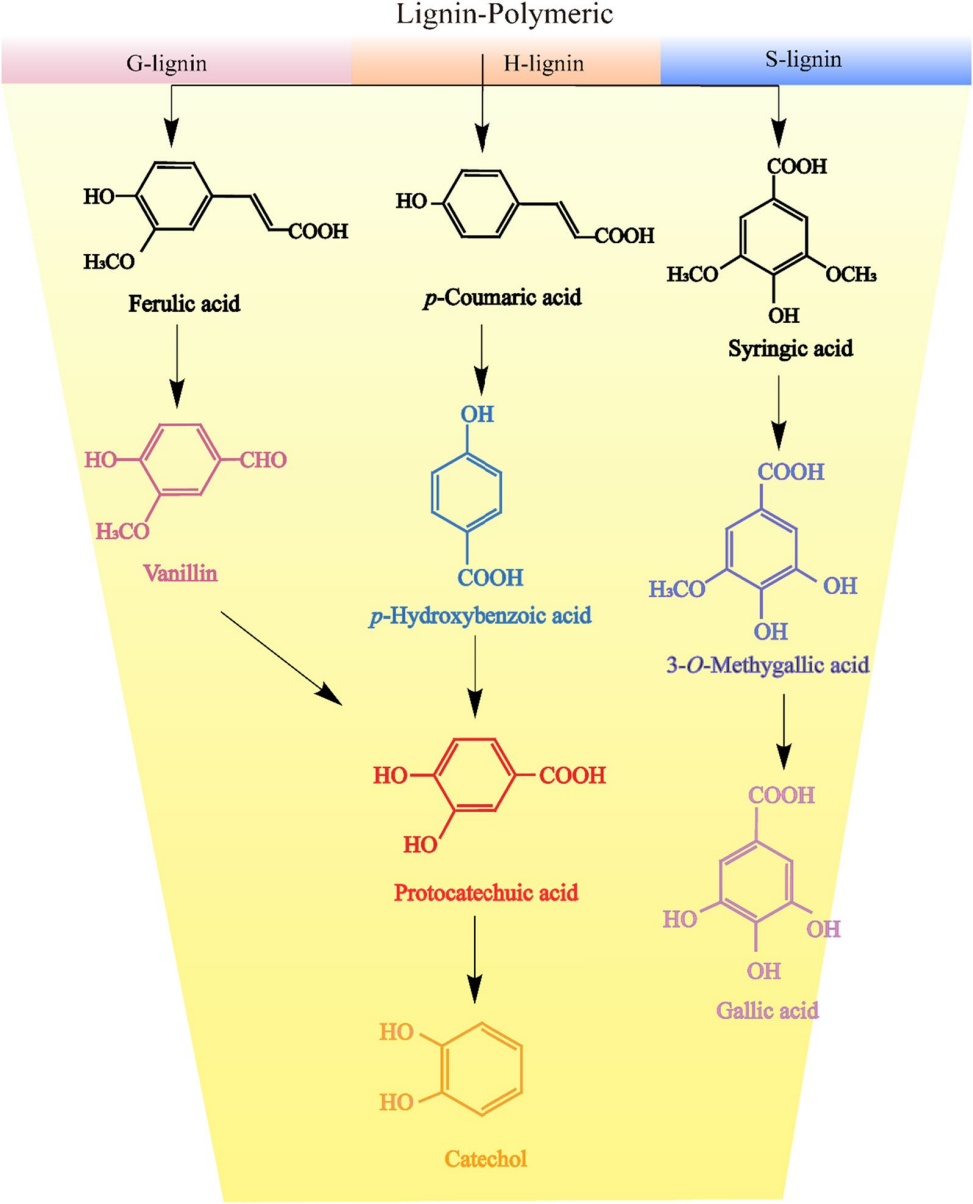
Lignin conversion into aromatics in “biological funnel” form

Lignin is considered an alternative source for the production of various polymers and biomaterials, not only as a raw material that can be converted into valuable products by biocatalysts, but also offering certain biotechnology applications. For example, genetic engineering strategies can be combined with biosynthetic pathways for high-value lignin products, lignin-derived materials for biotechnology applications (biosensors of lignin), as well as lignin-based hybrid materials for biomedical applications, including drug delivery, wound healing, and tissue engineering [123]. Recently, the conversion of lignin into high-value products by “biocatalysts” has become the subject of extensive research. The microbial degradation of lignin can produce a heterogeneous mixture of aromatic monomers, serving as a carbon and energy source for bacteria. During lignin depolymerization, three phenylpropane structural units, namely, guaiacyl (G), syringyl (S), and hydroxyphenyl (H), serve as starting points through upstream channels to produce central aromatic intermediates, such as protocatechuic acid, catechol, and 3-*o*-methylgallic acid or GA. Some valuable aromatic compounds will be produced, such as vanillin, *p*-hydroxybenzoic acid, and GA. Protocatechuic acid or catechol will be converted into a high-value product through a downstream route [124]. The process of metabolizing lignin-derived aromatics into specific compounds is known as “biological funnel”, which maximizes lignin utilization (Fig. 2) [125].

### Bacterial catabolic pathways of lignin into biochemicals

#### Vanillin synthesis and metabolic pathways

**Fig. 3:**
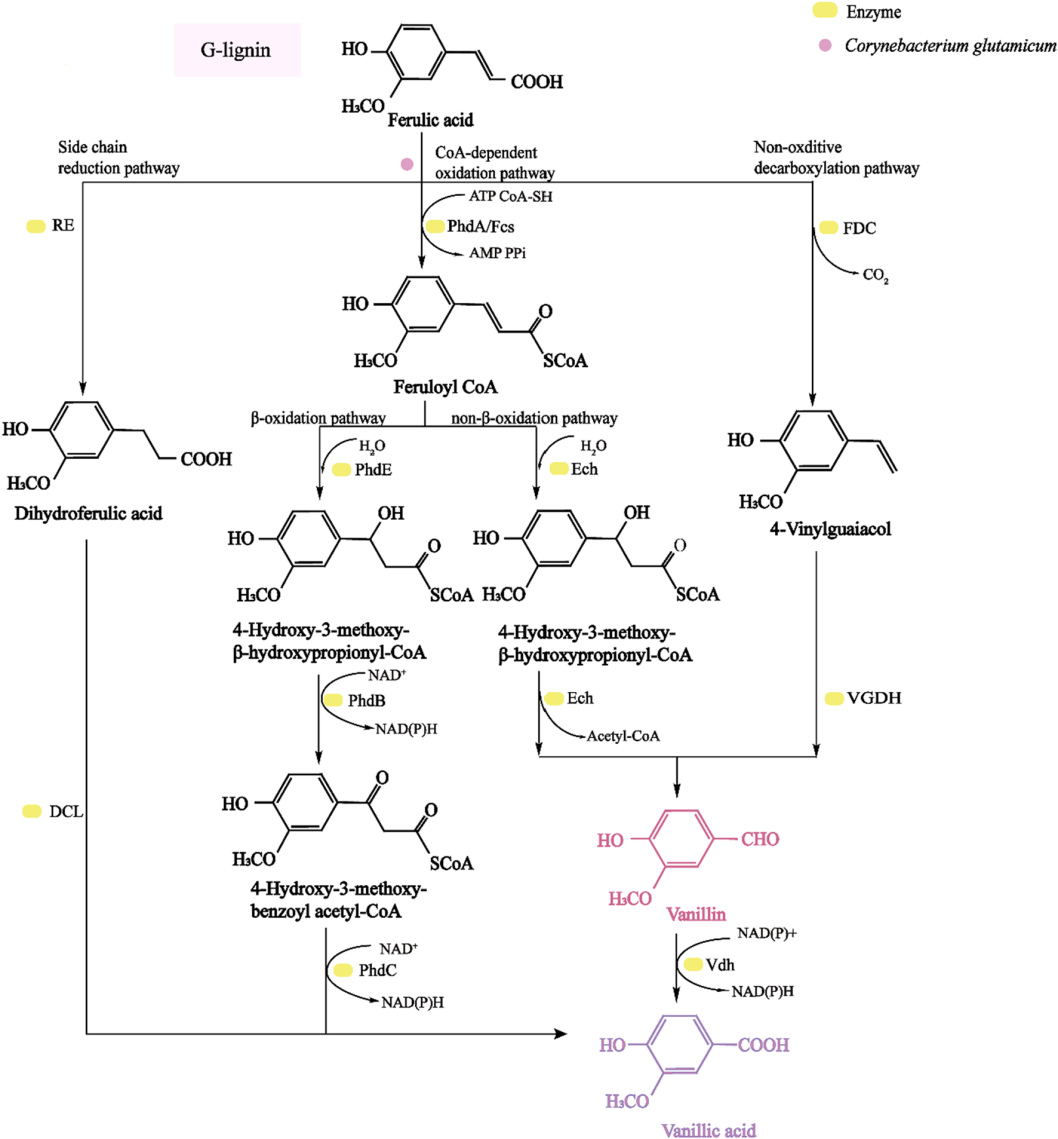
Vanillin synthesis and metabolic pathways

Vanillin (4-hydroxy-3-methoxybenzaldehyde) serves as an important biochemical substance produced by the bacterial degradation of lignin. Vanillin is a widely used edible spice that can serve as a plant growth promoter, fungicide, and oil defoamer, consisting of a high-value chemical product and as an important intermediate in lignin depolymerization. According to Mordor Intelligence, global vanillin revenue reached $428.46 million USD in 2019, which is expected to grow at a compound annual rate of 5.8% between 2021 and 2026 (https://www.mordorintelligence.com/). The rapid growth of utilizing products developed with natural ingredients has created strong growth opportunities for the global natural vanillin market. Vanillin can be isolated as an intermediate during biological lignin decomposition, and is produced through ferulic acid, eugenol, or isoeugenol conversion pathways [126]. Ferulic acid represents lignin-derived G-aromatics, with G-type lignin units are abundant in biomass, accounting for more than 95% of softwood [108]. The ferulate-mediated pathway is typical for vanillin production (Fig. 3), with vanillin produced by ferulic acid through CoA-dependent non-β oxidation and non-oxidative decarboxylation [127]. In *Corynebacterium glutamicum*, ferulic acid will be transformed by PhdA to ferulic coenzyme A, which will then be catalyzed by enoyl-CoA hydratase (Ech) to form the hydroxyl groups. The hydroxyl group will then be directly cleaved by Ech to form vanillin and acetyl-CoA. In the non-oxidative decarboxylation pathway, ferulic acid will be transformed to produce 4-vinyl guaiacol by decarboxylase, with vanillin further transformed by vinylguaiacol dehydrogenase (VGDH). Vanillin can be transformed by vanillin dehydrogenase (Vdh) to produce vanillic acid. Vanillic acid is a metabolite of vanillin and a necessary intermediate for the conversion of vanillin into protocatechuic acid. Vanillic acid can be produced through the vanillin pathway, CoA-dependent β-oxidation, and the side-chain reduction pathway. As shown in Fig. 3, ferulic acid can be transformed by PhdA to ferulic CoA, which will then be further transformed by enoyl-CoA hydratase/aldolase (PhdE). The intermediate with the hydroxyl groups can be oxidized by PhdB to produce a keto group, and the keto group will be subsequently cleaved by PhaC to produce vanillic acid and acetyl-CoA. The side-chain reduction pathway occurs mainly in anaerobic bacteria, with ferulic acid is transformed by aromatic reductase (RE) to form dihydroferulic acid, followed by decarboxylase (DCL) to form acetic acid and vanillic acid [128].

The ability of wild-type microorganisms to decompose lignin into vanillin remains limited, though metabolic engineering can improve the vanillin-synthesis efficiency. Studies have shown that mutant strain *R. jostii* RHA1 can produce 96 mg/L vanillin using lignin when cultured on 2.50% wheat straw biomass [129]. *P. putida* KT2440 can convert 86% of ferulic acid into vanillin [126], and microbial metabolic engineering was reported to increase vanillin production in several microorganisms [38]. After fermentation in the presence of wheat straw for 144 h, the deletion of *Vdh* gene in *R. jostii* increased vanillin yield to 96 mg/L [129].

#### *p*-Hydroxybenzoic acid synthesis pathway

**Fig. 4:**
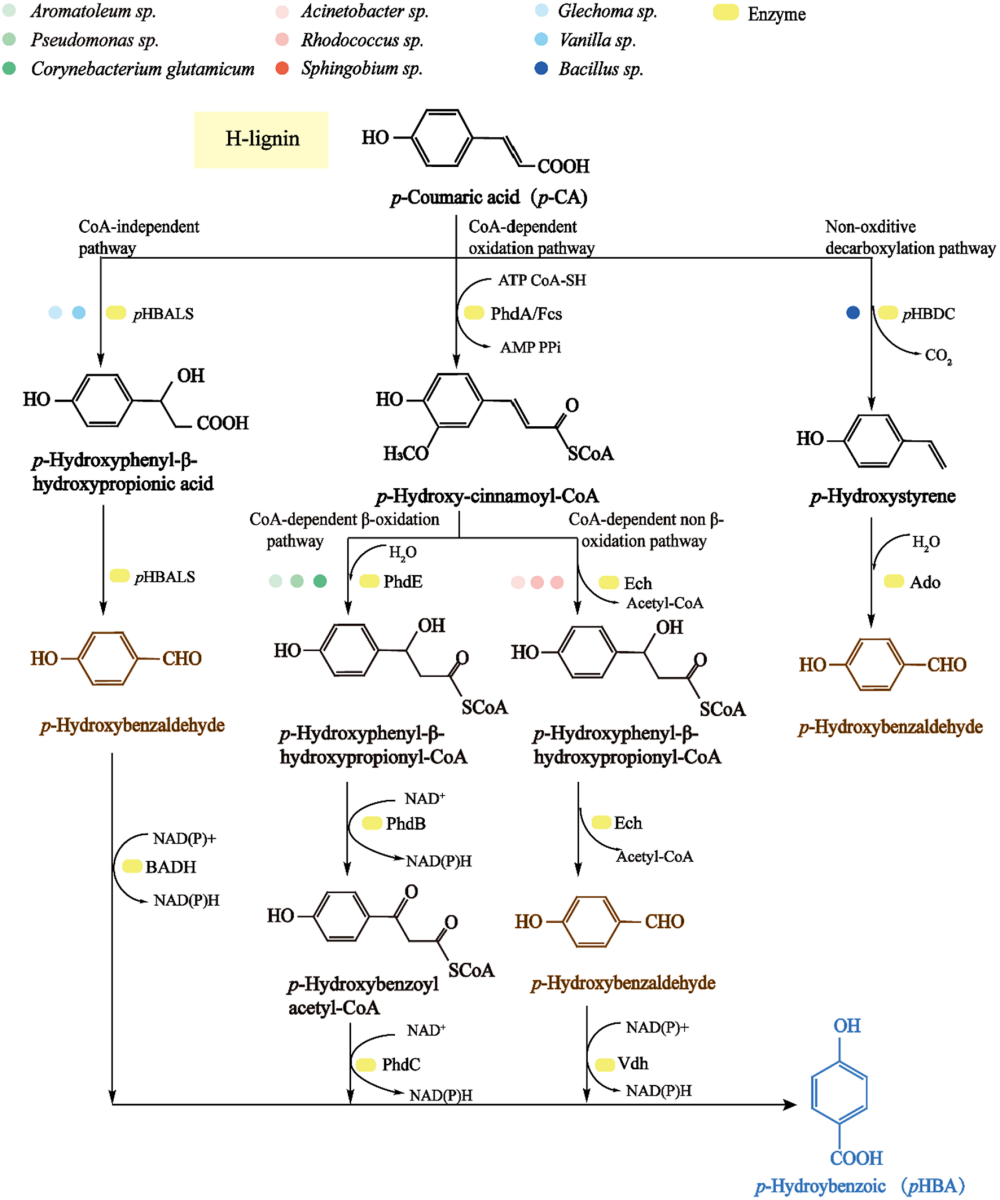
*p*-Hydroxybenzoic acid synthesis pathways

*p*-Hydroxybenzoic acid (*p*HBA) serves as an important central product in the degradation of H-type lignin, with strong antibacterial properties due to its phenolic hydroxyl structure. According to Global Info Research (GIR) statistics, the global revenue of parabens was approximately $85 million USD in 2021 and is expected to reach 100.8 million USD in 2028. Moreover, the compound annual growth rate is expected to reach 2.4% between 2022 and 2028 (https://www.globalinforesearch.com.cn/). With increasing market demand for *p*HBA, the use of renewable resources, instead of petroleum, as raw materials to produce *p*HBA has become a global priority. *p*HBA forms from H-type lignin aromatics [a representative substance being *p*-coumaric acid (*p*-CA)], with the existence of four types of metabolic pathways (Fig. 4). The CoA-dependent β oxidation pathway of *p*-CA usually occurs in certain deformed bacteria, such as *Aromatoleum* sp., *Pseudomonas* sp. and *C. glutamicum* [130]. This pathway will be transformed by PhdA and produce hydroxycinnamoyl-CoA thioester. The hydration of the *p*-CA double bond of thioester will be transformed by enoyl-CoA hydratase (PhdE) to produce hydroxyl groups. The intermediate with the hydroxyl group will then be oxidized by PhdB to produce a keto group that can be subsequently cleaved by PhaC to produce benzoyl-CoA and acetyl-CoA, producing *p*-hydroxybenzoic acid. The CoA-dependent non-β-oxidation pathway exists in *Acinetobacter* sp., *Pseudomonas* sp., *Rhodococcus* sp. and *Sphingobium* sp.[131]. The first two steps of this pathway in *Pseudomonas* sp. were found to be identical to the CoA-dependent β-oxidation pathway. The non-β oxidation pathway uses feruloyl-CoA synthetase (Fcs) and enoyl-CoA hydratase/aldolase (Ech) to transform* p*-CA and form a hydroxyl group, which can be directly cleaved by Ech to form *p*-hydroxybenzaldehyde and acetyl-coenzyme A. *p*-hydroxybenzaldehyde can then be transformed by Vdh to form *p*-hydroxybenzoic acid. The CoA-independent pathway of H-type lignin aromatics in *Glechoma* sp. and *Vanilla* sp. shows a similar conversion process, forming *p*-hydroxyphenyl-β-hydroxypropionic acid without forming CoA thioesters. Furthermore, *p*-hydroxyphenyl-β-hydroxypropionic acid will be oxidized to *p*-hydroxybenzoic acid [132]. The decarboxylated pathway in *Bacillus* sp. *p*-hydroxybenzoic acid decarboxylase (*p*HBDC) can also directly convert decarboxylase *p*-CA to *p*-hydroxystyrene and CO_2_, and aromatic dioxygenase (Ado) can transform *p*-hydroxy styrene to *p*-hydroxybenzaldehyde. This pathway has been used to produce *p*-hydroxystyrene.

Jung et al. reported on the engineering of a natural pathway for converting *p*-CA into *p*-hydroxybenzoic acid (*p*HBA) to realize a > 100-fold increase in the amount of accumulated *p*HBA. *Burkholderia glumae* BGR1 can be efficiently grown as a sole carbon source on *p*-CA via the CoA-dependent non-β-oxidation pathway. In this pathway, two carbon atoms were removed from *p*-CA as acetyl-CoA to produce *p*-hydroxybenzaldehyde, which will be subsequently oxidized to *p*HBA. The Palk promoter was used to express PHCS II in the *p*-CA batch reaction at 20 mM, resulting in a *p*HBA conversion rate of 99.0% [133].

#### Synthesis and metabolic pathway of protocatechuic acid

**Fig. 5:**
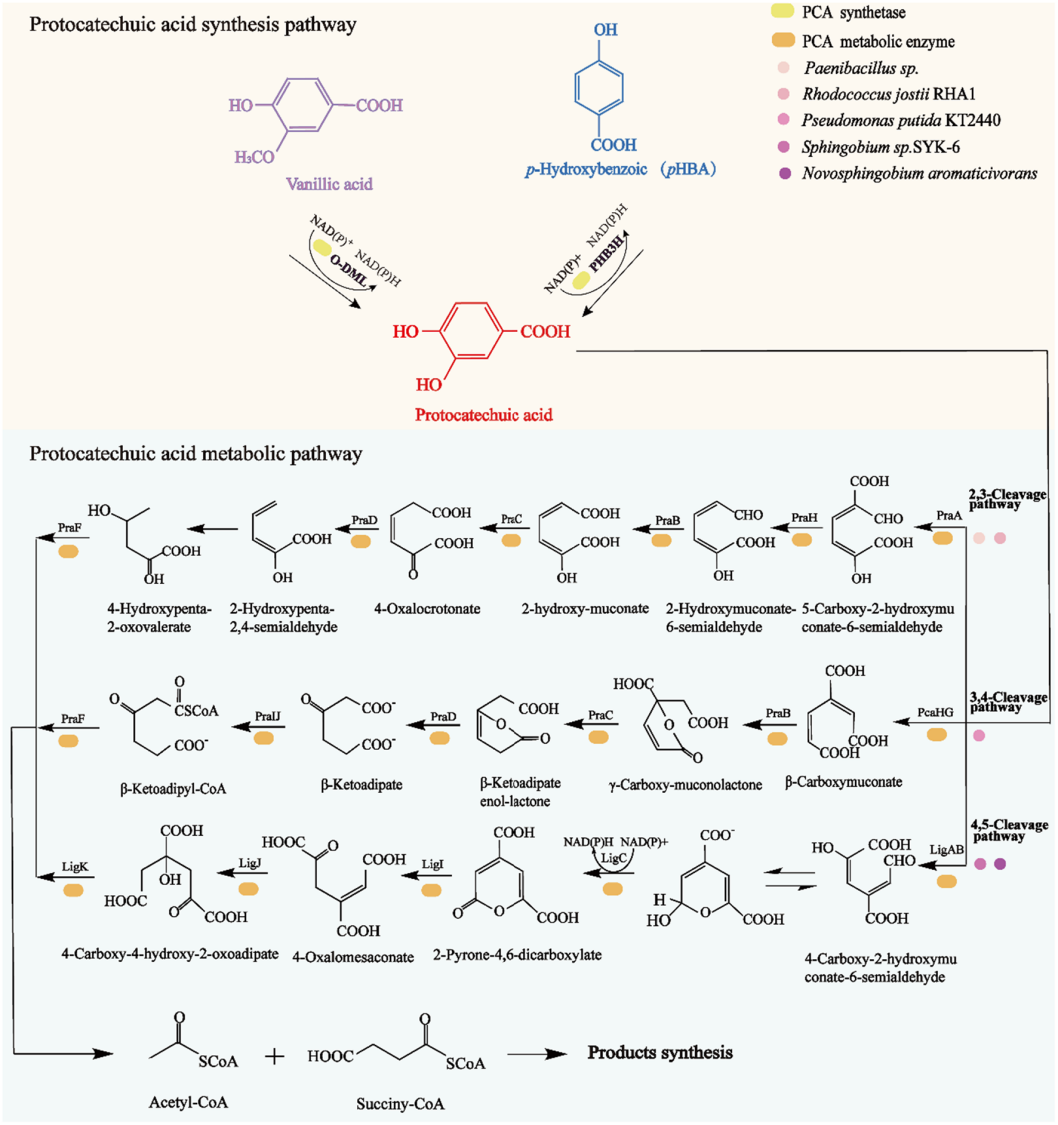
PCA synthesis and metabolic pathways

Protocatechuic acid (PCA), also known as 3,4-dihydroxybenzoic acid, is one of the important central aromatic intermediates in the process of bacterial degradation of lignin, serving as a high-value chemical product that exists in many vegetables and fruits, and also an effective active component in many Chinese medicines (such as *Salviae miltiorrhizae* and folium hibisci mutabilis). According to GIR statistics, the global revenue of protocatechuic acid in 2022 was about 13 million USD, with an annual compound growth rate is expected to reach 3.4% from 2023 to 2029 (https://www.globalinforesearch.com.cn/). Protocatechuic acid has a large market development space, with the annual market demand growth rate expected to remain at about 10%. As an important connecting platform between upstream and downstream pathways, protocatechuic acid plays an important role in the process of lignin depolymerization. Lignin-derived G-aromatics can be transformed into vanillic acid through the ferulic acid pathway (Sect. “Vanillin synthesis and metabolic pathways”), and then transformed by *o*-demethylase (O-DML) to produce PCA. The aromatic hydrocarbon derived from H-type lignin, i.e., *p*-CA will generate *p*-hydroxybenzoic acid through three pathways and then further generate PCA under the transformation action of 4-hydroxybenzoate 3-monooxygenase (pobA) (Fig. 5).

There are three PCA cleavage pathways, namely, the 2,3-cleavage pathway, 3,4-cleavage pathway (β-ketoadipic acid pathway) and 4,5-cleavage pathway (Fig. 5) [134]. *Paenibacillus* sp. contains the a 2,3-cleavage pathway, in which PCA initially transforms by protocatechuic acid 2,3-dioxygenase and subsequently converts into 4-hydroxy-2-oxovalerate by PraH, PraB, PraC, PraD, and PraE (2,3-cleavage pathway genes) [135]. The *R. jostii* RHA1 Vdh deletion mutant will produce a small amount of vanillic acid when grown on a medium containing wheat straw lignocellulose. By overexpressing protocatechuic acid 3,4-dioxygenase in engineered mutant *R. jostii* pTipQ2-praA, 2,5-pyridine dicarboxylic acid has been successfully produced using vanillin as the carbon source[27]. PCA was initially transformed by protocatechuic acid 3,4-dioxygenase and subsequently though PcaB, PcaC, PcaD and PcaI/J to produce β-ketoadipic acid in *P. putida* KT2440 [118]. β-ketoadipate was further cleaved by PcaF to produce succinyl-CoA and acetyl-CoA [136]. Acetyl-CoA produced by this pathway has also been used to synthesize a variety of compounds, such as PHA, lipids, itaconic acid and other chemicals [137]. The 4,5-cleavage pathway can initially convert PCA into 4-carboxyl-2-hydroxy-muconic acid-6-hemialdehyde (CHMS) by protocatechuic acid 4,5-dioxygenase (LigAB) in *Sphingobium* sp. SYK-6 and *Novosphingobium aromaticivorans *[138]. CHMS will be automatically converted into intramolecular hemiacetal form, which will then be oxidized by CHMS dehydrogenase (LigC) to produce 2-pyrone-4,6-dicarboxylic acid (PDC). LigI/J will further transform the PDC to produce 4-carboxyl-4-hydroxy-2-oxoadipate (CHA), which can be metabolized by CHA aldolase (LigK) to produce pyruvate and oxaloacetic acid. Studies have shown that *P. putida* KT2440 will adopt the 4,5-cleavage pathway design, with LigABC inserted into *P. putida* KT2440 instead of pcaHG, mediating cleavage from PCA to CHMS. LigC can be used to further mediate the conversion of CHMS into PDC, significantly improving the conversion of aromatics [139, 140].

#### Catechol synthesis and metabolism pathway

**Fig. 6:**
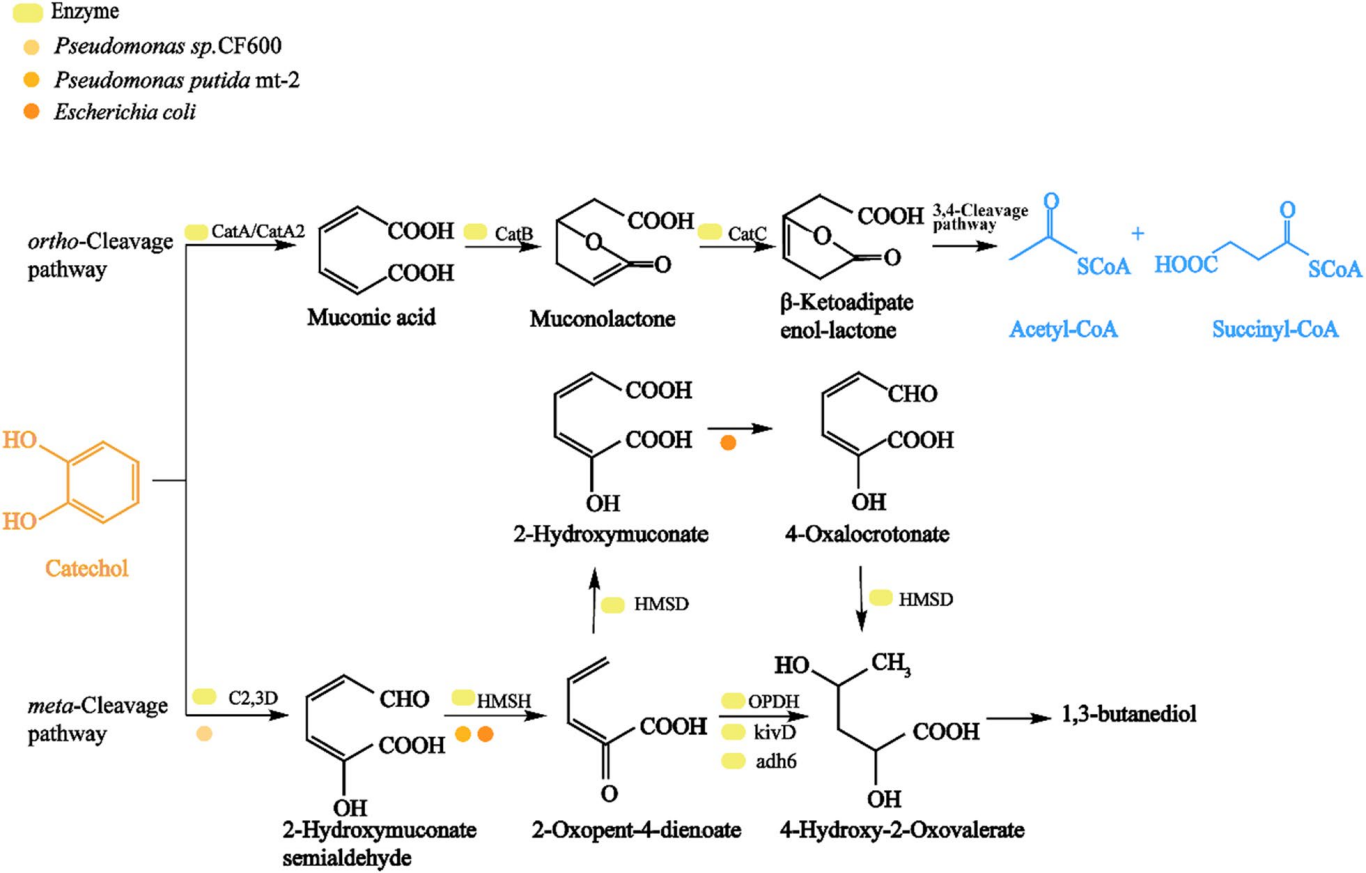
Metabolic pathways of catechol

Catechol is also an important central intermediate, and because of its functional diversity, as well as use in dyes, pesticides, photosensitive materials, and plating materials. According to GIR, the global catechol revenue in 2022 was approximately $128.9 million USD, with an expected compound growth rate of 3.4% from 2023 to 2029 (https://www.globalinforesearch.com.cn/). The application field of tea polyphenols continues to increase, with growing downstream market demand. Overall, certain supply gaps remain in the tea phenol market. Catechol, a platform intermediate, undergoes the lignin depolymerization progress. PCA decarboxylase (AroY) can further transform the production of catechol from PCA, and downstream products can be obtained through the ortho-cleavage and meso-cleavage pathways in bacteria [141]. The ortho-cleavage pathway can convert catechol into cis-muconic acid (Fig. 6) [142]. In addition, cis-muconic acid can be transformed by catalase (CatB) and CatC to produce β-ketoadipate enol-lactone, regenerating into succinyl-CoA and acetyl-CoA. The meso-cleavage pathway of catechol will be transformed by catechol 2,3-dioxygenase (C23O) to produce 2-hydroxymuconic semialdehyde, which can be found in *Pseudomonas* sp. CF600. Some studies have successfully produced 1,3-butanediol from lignin-derived aromatics in recombinant *E. coli* by constructing the intermediate-cleavage pathway of catechol. Through the heterologous expression of xylEFJ in *P. putida* mt-2 and mhpD in *E. coli*, engineered *E. coli* produced 4-hydroxy-2-oxopentanoate via the meta-cleavage pathway of catechol. The main process behind this was that 2-hydroxy-muconic acid semialdehyde, which generated an intermediate 2-oxovalerate-4-dienoic acid ester through muconic acid semialdehyde hydrolase (HMSH), before further generating 4-hydroxy-2-oxovalerate ester through propane oxidative dehydrogenation (OPDH). Furthermore, the kivD of *Lactococcus lactis* and adh6 of *Saccharomyces cerevisiae* were introduced into recombinant *E. coli* to further synthesize 1,3-butanediol [119]. In addition, 4-hydroxy-2-ketovalerate aldolase (MhpE) and acetaldehyde dehydrogenase (MhpF) can be introduced into *E. coli*, and intermediate 2-oxovalerate 4-dienoate can be converted into 2-hydroxy-muconic ester through semi-dehydrogenase, which can be further converted into pyruvate and acetyl-CoA (Fig. 6).

#### Synthesis pathway of cis-muconic acid

Cis-muconic acid is a typical dicarboxylic acid, which has attracted significant attention as a precursor for the production of isophthalic acid [143]. According to GIR statistics, global muconic acid revenue in 2021 was about 40 million USD, and with a compound annual growth rate is expected to reach 2.6% from 2022 to 2028 (https://www.globalinforesearch.com.cn/). The increasing incidence of packaged goods and beverages in food is expected to further increase the demand for plastic packaging, thus, muconic acid will likely further drive the market growth. Bacteria can catabolize lignin-derived aromatics to cis-muconic acid through the β-ketoadipic pathway and catechol ortho-cleavage pathway. In *P. putida*, cis-muconic acid can be obtained by inactivating muconate cycloisomerase (CatBC) and exogenously expressing phenol monooxygenase or guaiacol *o*-demethylation in the catechol branch pathway. Through the deletion of pcaHG and overexpression of PCA decarboxylase from *Enterobacter cloacae*, cis-muconic acid has been effectively synthesized through a PCA (Fig. 6) [131].

The conversion of ligno-derived aromatics to cis-muconic acid with high efficiency has been realized through genetic engineering and fermentation technology. CatA was overexpressed in *C. glutamicum* by inactivating CatBC and using the fed-batch fermentation strategy, with a high yield of PCA (85 g/L) cis-muconic acid was thus obtained [144]. By deleting CatB in *Myxomatosis* sp. ATCC 39116, cis-muconic acid was successfully synthesized with a titer of 1.96 g/L and a yield of 24% [143]. In addition, the high-efficiency synthesis of muconic acid was achieved by combining genetic engineering technology with feeding batch technology. For example, the endonuclease coding genes *endA-1* and *endA-2* were deleted in *P. putida* KT2440 MA-9, and the catechol 1,2-dioxygenase and phenol hydroxylase genes *dmpKLMNOP* were overexpressed. Thus, the strain converted the pyrolyzed lignin into muconic acid, which could be further generated into adipic acid after hydrogenation. In addition, polycondensation with hexamethylenediamine could result in the formation of the first nylon derived from lignin [145].

#### Synthesis and metabolic pathway of GA

**Fig. 7:**
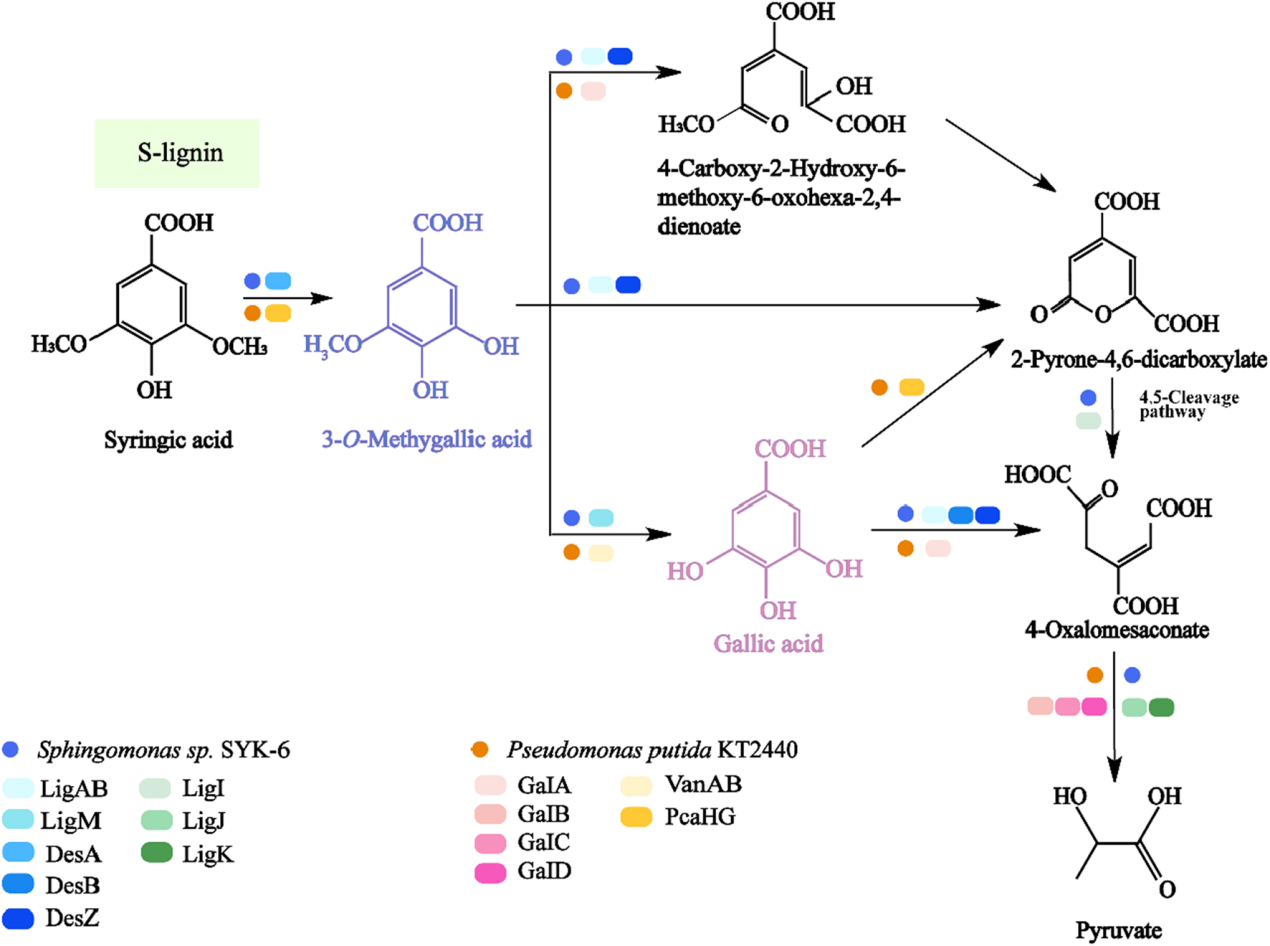
Gallic acid synthesis and metabolic pathways

GA is mainly used in the food, medicine, biology, agriculture and chemical industries. According to the statistics of GIR, the global GA revenue in 2022 was about 79 million USD, with its compound annual growth rate expected to reach 7.5% from 2023 to 2029 (https://www.globalinforesearch.com.cn/). China is the largest GA market, accounting for about 86% of the market share. Moreover, GA raw materials come from various sources, with GA exhibiting excellent biological activity. Thus, a wide range of applications can be obtained, resulting in high demand for GA by the Chinese market. GA is also an important and valuable intermediate product in the lignin degradation pathway. GA can be usually obtained in the depolymerization of S-type lignin, with representative S-derived aromatic compounds consisting of syringic acid [146]. Degradation of syringic acid by tetrahydrofolate-dependent *o*-demethylase (DesA) will produce 3-*o*-methylgallic acid (3MGA) in *Sphingomonas* sp. SYK-6. Subsequently, 3MGA can be catabolized through two pathways (Fig. 7). In the assimilation pathway, 3MGA will further be *o*-demethylated by LigM to produce GA. In the degradation pathway of syringic acid, 3MGA and GA serve as two central intermediates. *Sphingomonas* sp. SYK-6 were originally transformed 3MGA by using 3-*o*-methylgallate 3,4-dioxygenase (DesZ) or LigAB to produce 2-pyranone-4,6-dicarboxylate. 2-Pyranone-4,6-dicarboxylate can be integrated into the 4,5-cleavage pathway of PCA and cleaved by LigI to produce 4-oxalketol [147]. As a key intermediate, GA can be converted into 4-oxalic acid by gallate dioxygenase (DesB) or LigAB, followed by the yield of 4-carboxyl-4-hydroxy-2-oxoadipate (CHA) transformed by LigJ. LigK can cleave CHA, producing pyruvate and oxaloacetic acid [148]. Furthermore, by introducing LigAB into *Pseudomonas putida* KT2440 and overexpressing vanillate o-demethylase (VanAB), the engineered strain can convert syringic acid to yield 2-pyranone-4,6-dicarboxylic acid (Fig. 7) [140]. In order to achieve the synthesis of GA from *p*-coumaric acid, VanAB can be replaced by a two-component flavin-dependent monooxygenase (HpaBC) of *E. coli.* Under optimal conditions, 20 mM *p*-coumaric acid can produce 19.96 mM GA, with a conversion rate of nearly 100% [149]. Therefore, the synthesis of new intermediates can be explored in the assimilation pathway of lignin-derived aromatics, serving as a new method to produce a wide range of valuable chemicals.

#### Synthetic and metabolic pathways of other products

**Fig. 8:**
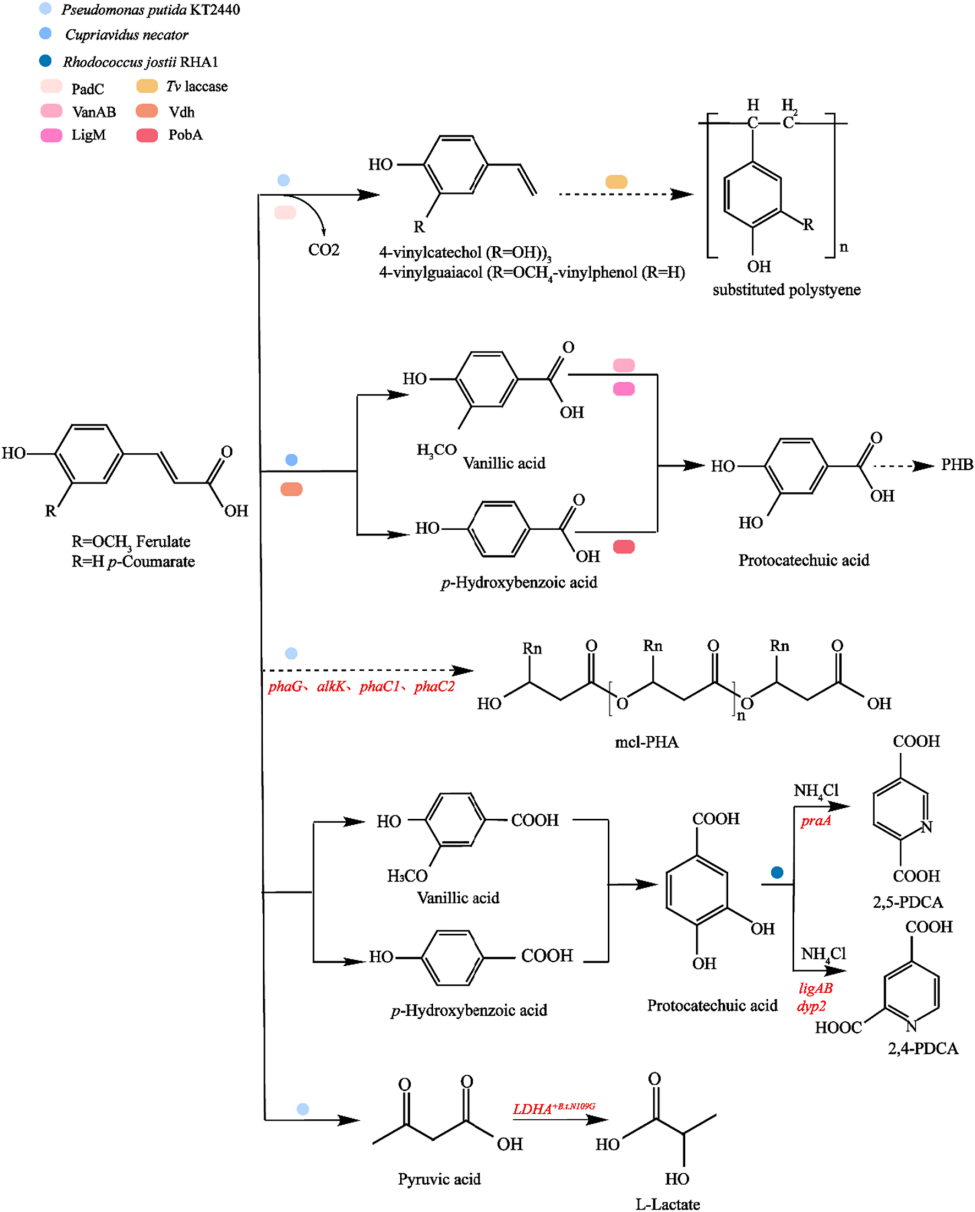
Synthetic and metabolic pathways of other products

In addition to the above products, lignin can also be converted into other biological products (Fig. 8), such as 4-vinylguaiacol, polyhydroxybutyrate (PHB), polyhydroxyalkanoate (mcl PHA), L-lactic acid, pyridine-2 and 4/5-dicarboxylic acid (2,4/5-PDCA). Specifically, 4-vinylguaiacol is commonly used by the spice industry, and in pharmaceutical products, with a market value is about 40 times that of ferulic acid [150]. It can be further converted into a variety of high-value products, such as ethyl guaiacol and vanillic acid. PHB exhibits biodegradable properties and broad application prospects in medical materials, degradable plastics, and sewage treatment. In terms of production, microbial fermentation is the main production process of PHB [151]. mcl-PHA is a naturally produced polymer with valuable properties, though commercial development remains limited due to the high costs associated with its production and extraction. l-Lactic acid is mainly used in the food, medicine and pesticide industries. According to IHSMarkit data, global lactic acid market consumption in 2018 was about 518,000 tons. Over the next 5 years, lactic acid consumption is expected to experience a 5.6% compound annual growth rate (https://global.ihs.com/standards.cfm?publisher=IHS). 2,4-PDCA and 2,5-PDC, as dicarboxylic acids, can be used as building blocks for aromatic polymers (including polyamides and polyesters), and serve as target chemicals in bio-based plastics [108].

Williamson et al. [126] replaced the Ech in *P.putida* KT2440 by integrating phenolic acid decarboxylate (PadC) from *Bacillus subtilis* into hydroxy-cinnamic acid-induced ferR operon. Subsequently, *p*-CA and ferulic acid were converted to 4-vinylguaiacol and 4-vinylphenol, respectively, while 4-vinylguaiacol and 4-vinylphenol were biopolymerized using laccase from *Trametes versicolor* to produce “biostyrene” materials on a small scale.

Moreover, Weng et al. [152] designed and constructed in *Cupriavidus necator* a pathway that integrated an endogenous CoA-dependent non-β oxidation pathway and its primitive hydroxybenzoic acid metabolism, which could convert ferulic acid and *p*-hydroxybenzoic acid into PHB. Ferulic acid and coumaric acid were then catabolized to vanillic acid and *p*-hydroxybenzoic acid under the catalysis of Fcs, Ech and Vdh enzymes, then further transformed into the intermediates of protocatechuic acid through the expression of VanAB or LigM and endogenous *p*-hydroxybenzoic hydroxylase (PobA). Then, they are transformed into succinyl-CoA and acetyl-CoA via the endogenous β-ketoadipic pathway. Acetyl-CoA can then be metabolized to produce PHB.

Salvachua et al. [153]used genetic engineering technology to integrate *phaG, alkK, phaC1*, and *phaC2* in *P. putida* KT2440 into chromosomes, and the overexpression of the constitutive Ptac promoter was used to enhance the transformation of *p*-CA into polyhydroxy-chain alkanoate (mcl-PHA). The yield (g mcl-PHA per of cell dry weight) increased by 20%.

Johnson et al. [141] synthesized a gene encoding *Bacillus* lactate dehydrogenase *LDHA* and added the amino acid substituting group N109G, which was expressed in *P. putida*. The study found that pyruvate could be aerobically converted into l-lactic acid by binding the lactate dehydrogenase coding gene *LDHA*^+B.t.N109G^.

Spence et al. [154] also overexpressed the ligAB of *Sphingobium* sp. SYK-6 and the *dyp2* gene of *Amycolatopsis* sp.75iv2 in *R. jostii* RHA1 (R. jostii ΔpcaHG:ligAB(Ptpc5)/pTipQC2-dyp2.), where the titer of pyridine-2, 4-dicarboxylic acid increased by more than 10 times. The *pcaHG* gene was replaced by the praA of *Paenibacillus.*sp, optimizing the synthesis of pyridine-2, 5-dicarboxylic acid.

### Bacterial catabolic pathway of lignin conversion into bioplastics

**Fig. 9:**
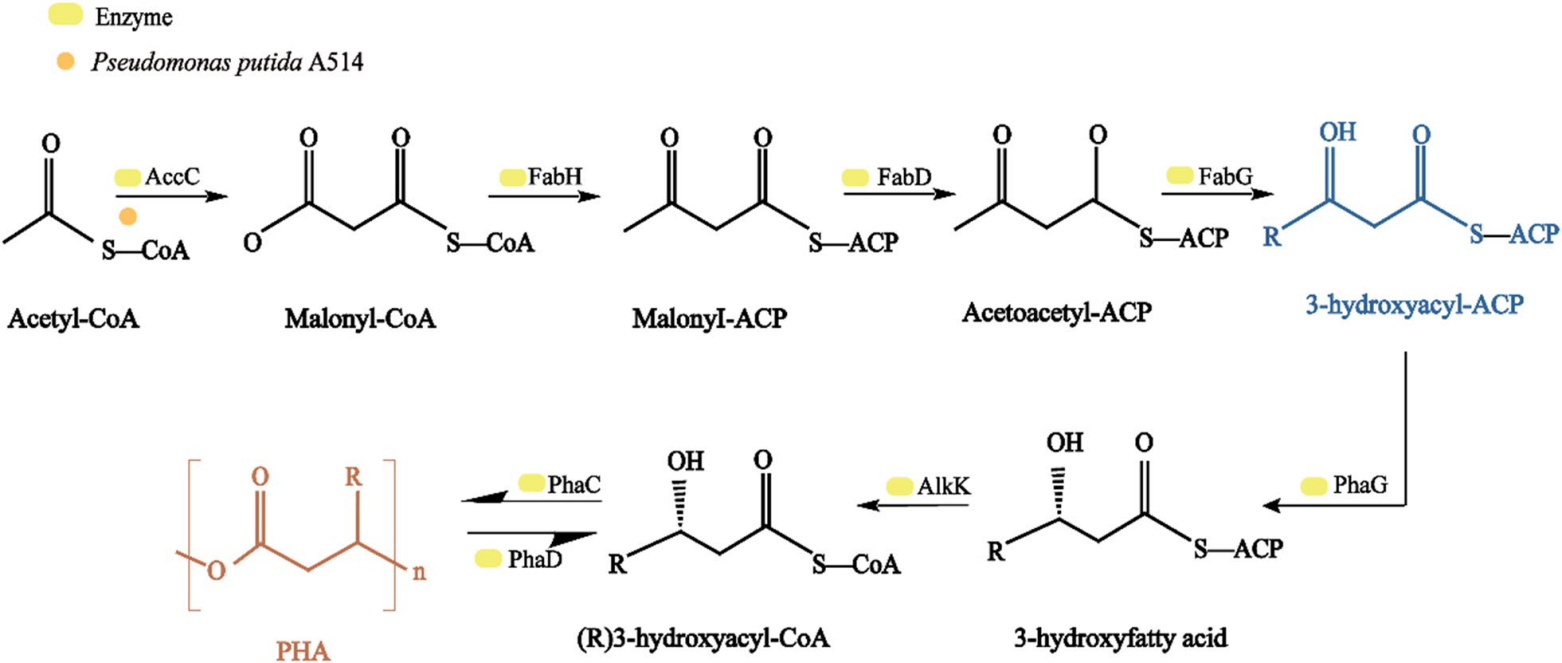
PHA biosynthesis pathways

Polyhydroxyalkanoate (PHA) is a promising bioplastic, with similar physical and chemical properties as fossil fuel plastics. Due to its biocompatibility and biodegradability, PHA can be used in various industrial fields, such as producing biomaterials and in medical applications [155]. According to Mordor Intelligence statistics, from 2023 to 2028, global polyhydroxy alkane acid ester (PHA) is expected to experience a 3% compound annual growth rate (https://www.mordorintelligence.com/). The main factor driving the market research includes the growing demand for environmentally friendly materials, and lignin-derived aromatics can be metabolized through the β-ketoadipic pathway to synthesize PHA in bacteria [37, 156]. Protocatechuic acid will then be converted from lignin-based aromatics into acetyl-CoA by ring cleavage, it has been described in detail in Sect. “Synthesis and metabolic pathway of protocatechuic acid”, as shown in Fig. 5. And then, acetyl-CoA is further decomposed into PHA [157]. The conversion of acetyl-CoA into PHA will be affected by β-ketothiolytic (phaA), acetyl-CoA reductase (phaB) and PHA synthase (phaC) in turn, with acetyl-CoA intermediate is converted into poly 3-hydroxybutyrate. Furthermore, the monomers of other short-chain-length (SCL) or medium-chain-length (MCL) PHAs will be synthesized by different metabolic pathways, such as the tricarboxylic acid cycle, beta oxidation and fatty acid biosynthesis pathways. The key enzyme includes phaC, which is involved in polymerization activity and controls the molecular weight and PHA monomer composition. Wang et al. [62] analyzed a key gene in *P. putida* A514 from the lignin monomer to the PHA synthesis pathway, and the study found that phaG (encoding 3-hydroxyl-ACP thioesterase) and alkK (encoding fatty acid-CoA ligase) were the key bridges connecting cell growth and PHA synthesis. These also played a role in PHA biosynthesis and cell growth, increasing the expression levels of phaG and alkK and improving both cell growth and PHA biosynthesis (Fig. 9).

It has been proved that *P. putida* can effectively convert aromatics into PHA. By integrating the three functional modules of lignin depolymerization, aromatic metabolism, and the above mentioned PHA synthesis pathway, with recombinant *P. putida* KT2440 consuming sulfate lignin and producing PHA [37]. These processes produce lignin components, which can be used for PHA synthesis by cleaving additional β–β and β-O-4 bonds, enriching G-type and H-type aromatics. The production of a large number of aromatic monomers will promote the synthesis of PHA lignin components. Moreover, Gram-negative soil bacterium *C. basilensis* can effectively degrade lignin and produce PHA [157]. These results indicated that the metabolically engineered bacteria can improve the catabolism of aromatics, promote the production of value-added products, and facilitate the transformation of lignin into PHAs during lignin transformation by bacteria.

### Bacterial catabolic pathway of lignin conversion into biodiesel

Lipids are a class of compounds found in nature that can easily dissolve in organic solvents. Their structural diversity can endow lipids with a variety of important characteristics, such as the ability to store energy, biofilm composition, and unique substances on the cell surface such as receptors and recognition factors. According to GIR statistics, the global biodiesel revenue in 2022 was about 24,720 million USD, with its compound annual growth rate is expected to reach 1.4% from 2023 to 2029 (https://www.globalinforesearch.com.cn/). Different countries accept biodiesel as mixtures, making biodiesel the fuel in the market. Lipids are produced by microbial species, with oil-producing microorganisms mainly consisting of bacteria, yeast, filamentous fungi and algae. Their oil production also considerably differs, with oil accumulation accounting for more than 20% of the dry weight of cells [158]. Moreover, oil-producing microorganisms exhibit different lipid production characteristics [159]. The biotransformation of lignin-derived aromatics into lipids was found to be relatively obvious in the gram-positive species of oleaginous *Rhodococci*, such as *Rhodococcus opacus*, which can produce intracellular lipids through the biodegradation of aromatic substrates. Acetyl-CoA intermediates obtained from lignin metabolism via the β-ketoadipate pathway have been integrated into the biosynthesis pathway of triacylglycerol, formed through the esterification of one glycerol molecule with three fatty acid chains [160].

**Fig. 10:**
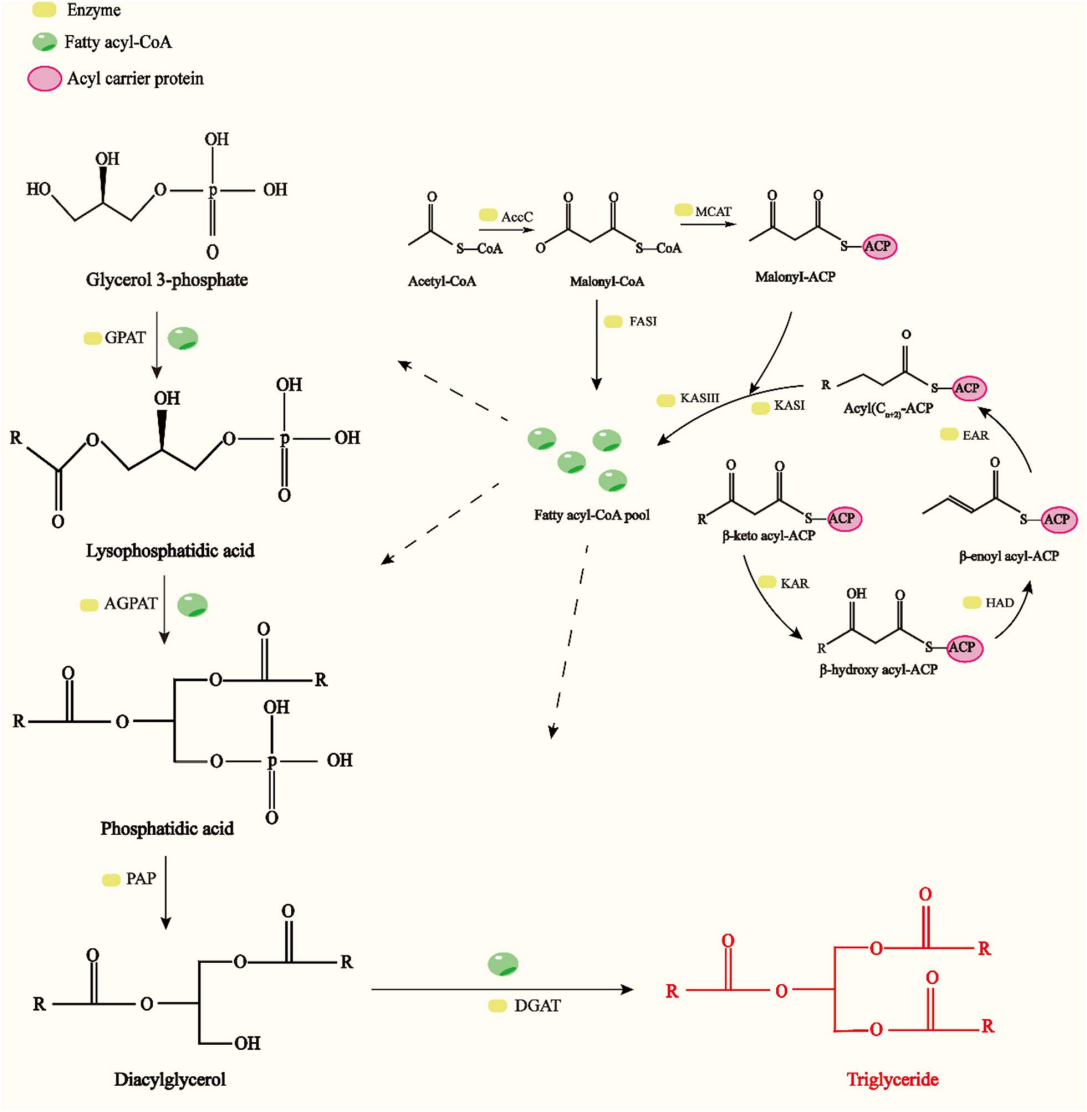
Lipid biosynthesis pathways

Oleaginous bacteria can degrade and catabolize lignin-derived aromatics and synthesize lipids from lignin. Kosa et al. [161] found that *R. opacus* DSM 1069 and PD630 could convert 4-hydroxybenzoic acid and vanillic acid, two lignin model compounds, into lipids under nitrogen-restricted conditions. Both strains could use 4-hydroxybenzoic acid and vanillic acid as carbon sources, accumulating about 20% lipids in their dry mass. For example, lipids have been produced by *R. opacus* PD630 successfully by using a range of pretreated lignin and some lignin models. The regulation of the laccase secretion pathway and lipid production module has made it possible to obtain a high concentration of lipid when transforming lignin-rich biological waste in *R. opacus* PD630 (Fig. 10) [162]. The aromatic compounds in lignin have also been transformed into acetyl-CoA, and used as a substrate for fatty acid synthesis, with lipid biosynthesis also representing the final step in lignin biotransformation in *R. opacus* PD630. FASI expression was found to be closely related to the rate of lipid accumulation, indicating that FASI is a key enzyme in oleaginous bacteria. Two types of fatty acid biosynthesis systems exist, with most bacteria only containing the type II system (FASII), using discrete and monofunctional enzymes to progressively perform decarboxylative Claisen condensation reactions, adding two carbons to each elongation step [163]. By contrast, FASI utilizes a single large, multi-unit, multifunctional enzyme for condensation and elongation to produce palmitic acid with greater efficiency. The type I system can be further integrated with type II enzymes to produce a greater variety of fatty acids. Therefore, the highly efficient FASI enzyme may explain the high lipid content realized by introducing acetyl-CoA into fatty acids in oleaginous bacteria such as *R. opacus* PD630.

### Bacterial catabolic pathway of lignin conversion into bioactive compounds

**Fig. 11:**
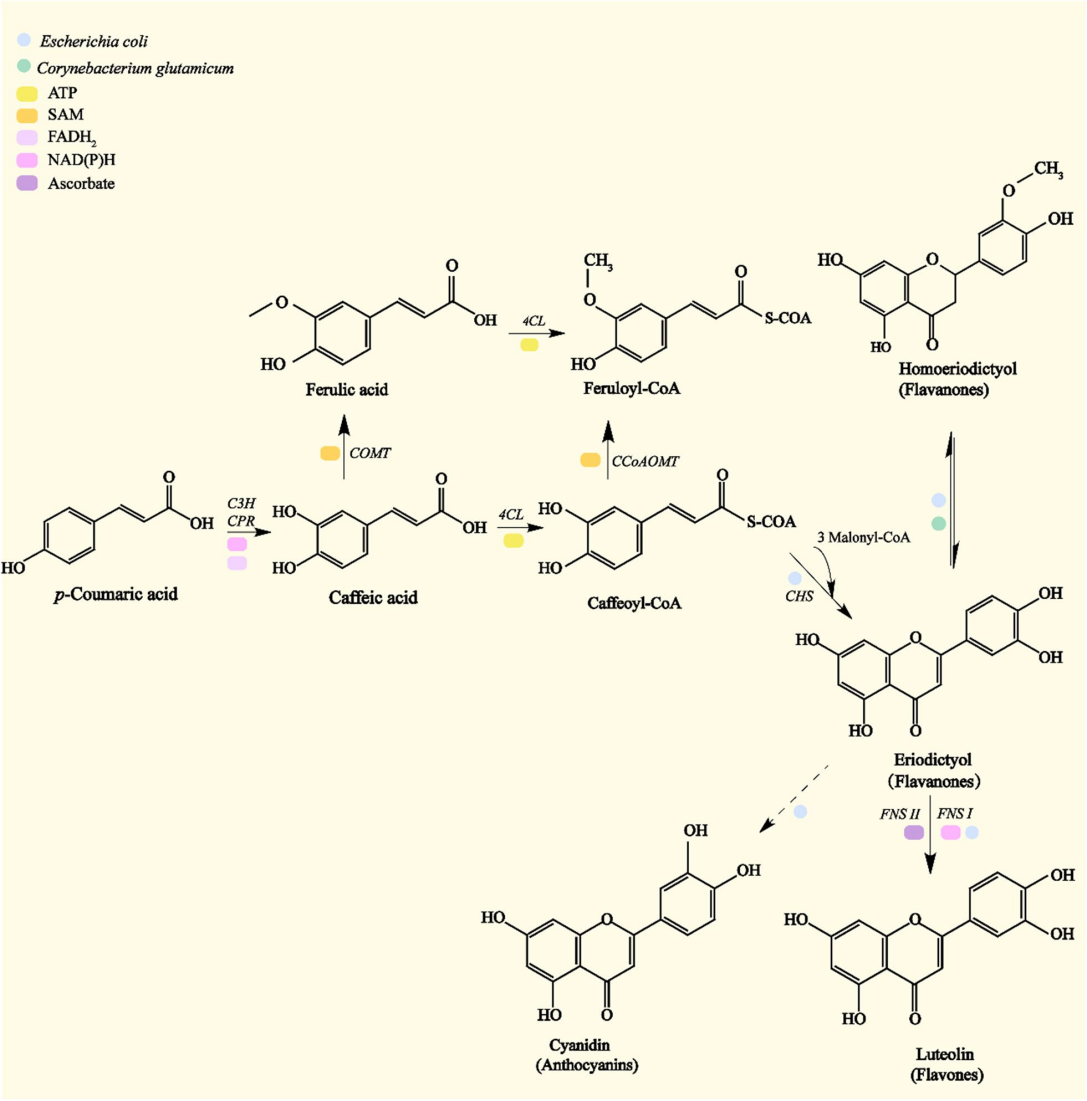
Biosynthetic pathways of flavonoids

Flavonoids are the most abundant secondary metabolites from plants, belonging to the polyphenol secondary metabolites family, and are widely used in health food, medicine and medical treatment. According to Mordor Intelligence, the flavonoids market is expected to grow at a compound annual growth rate of 3.9% from 2019 to 2024, with the flavonoid market reaching $1.2 billion USD by 2024 (https://www.mordorintelligence.com/). Anthocyanins, accounting for a major share of the global flavonoid market and are increasingly used as colorants in the food and beverage industry, and serve as the main drivers of growth. Due to the similarity of aromatic groups, microorganisms convert lignin-derived aromatic hydrocarbons into flavonoids and can promote the appreciation of lignin [164]. Lignin derivatives *p*-CA, ferulic acid, and caffeic acid can be used as major precursors along with other essential chemicals (acetyl-CoA and malonyl-CoA) in the flavonoid biosynthesis pathway (Fig. 11) [165].

Flavonoids transformed by lignin mainly include anthocyanins, flavanones flavonols, and flavones. Cyanidins are the most common product of anthocyanins, with the biosynthesis pathway of anthocyanins extending from the flavanol synthesis pathway through the expression of anthocyanin synthetase and glycosyltransferase. Cress et al. [166] constructed a pathway from catechins to O-methylated anthocyanins and increased the production of O-methylated anthocyanins to 56 mg/L using CRISPRI-mediated genetic engineering of *E. coli*.

Eriodictyol is a representative substance of flavanones, and studies have shown that caffeic acid can be used as a precursor. Through key enzyme selection, gene structure design, process optimization, and other specific strategies, researchers found that optimized *Escherichia coli* could react with caffeic acid to extract eriodictyol with a titer of 55 mg/L [167]. These results offer prospects for the efficient bioconversion of lignin-derived aromatic monomers into natural flavonoids.

Moreover, quercetin is a representative product of flavonols, with *p*-CA, caffeic acid, and ferulic acid consisting potential precursors for the biosynthesis of flavonols or flavanols. The core structure of polyphenols such as (2S)-flavanone naringin and stilbene resveratrol, can be synthesized by *p*-CA and caffeic acid, with flavanols and flavonols produced by expressing the exogenous dioxygenase gene, resulting in a quercetin titer of 10 mg/L [168]. Recombinant *E. coli* can convert quercetin from *p*-CA with a titer of 20 μg/L by simultaneously expressing six biosynthetases from alloplants, namely, 4CL, CHS, CHI, F3′5′ h, FHT, and FLS [169].

Luteolin is a common flavone product. Isoflavones are produced by the expression of isoflavone synthetases (IFS) and CPR in the flavonoid synthesis pathway. Flavonoids can be synthesized in one step using flavonoid synthase (FNS), instead of IFS. Through heterologous expression, the biosynthetic pathway of luteolin in *E.coli* and the synthetic pathway of pentanediol in sandalwood have been successfully constructed [170].

## Conclusions

This review summarized the lignin-degrading bacteria, lignin-degrading enzymes, and the catabolic mechanism of lignin degradation, enabling the high-value utilization of lignin. According to relevant studies, the high-value utilization of lignin was found to be closely related to bacteria and enzymes. By summarizing the pathways and genes involved in the catabolism of lignin and lignin-derived aromatics, a relatively complete lignin metabolic network could be identified. All the pathways of the lignin high-value process were discussed. The ability of lignin-derived aromatics to produce high-value chemicals and its environmentally friendly properties demonstrated that lignin may serve as a possible alternative to a variety of biological products and bioplastics.

Several studies have been conducted on the transformation of lignin by bacteria, with encouraging results, making major contributions toward achieving the microbial transformation of lignin. With the identification of an increasing number of lignin-degrading bacteria and degrading enzymes, there are more ways to transform lignin than before. Bacteria can upgrade lignin into valuable products through various processes, including lignin depolymerization, the uptake of aromatic products, and the catabolism of intracellular aromatic compounds.

## Challenges and prospects

Although remarkable progress has been made in the study of lignin high-value conversion, some problems remain to be solved in the process of lignin transformation by bacteria. First, the structure and types of lignin limit its transformation. The complex structure and high molecular weight of lignin limit its solubility in organic and inorganic solvents at room temperature, so it is difficult for bacteria to completely transform lignin. Second, lignin-transforming bacteria still require further exploration. At present, the genus of lignin-degrading bacteria is relatively simple, with most lignin-degrading bacteria only converting a portion of lignin-derived aromatics. Thus, the intermediate products generated by lignin conversion are also limited. Finally, the lignin-degrading enzyme system is not complete, and the mechanism is unclear. Although certain progress has been made in lignin-degrading enzymes in recent years, only a few studies have analyzed the synergistic effect of Lac, LiP and MnP [171]. However, gaps remain in the understanding of the synergistic mechanism of different types of lignin-degrading enzymes, with incomplete research on the protein interaction network among lignin-degrading enzymes, coenzymes and metabolic enzymes.

Therefore, great efforts are still needed to synthesize biological products and achieve a relatively complete metabolic network of lignin. In future studies, firstly, new processes should be introduced to decompose the substrate of lignin into precursors of low molecular weight and water-soluble precursor, which may assist the bacteria in transforming lignin into valuable products. The superior functions of different microorganisms also need to be integrated to regulate the lignin depolymerization process, improving the depolymerization efficiency of lignin and avoiding complete mineralization [172]. Second, bacteria that can convert lignin into value-added products need to be explored to achieve the efficient conversion of lignin, high-value intermediate types, and abundant synthetic pathways. Finally, new sources of lignin-degrading enzymes need to be further enriched, to provide high-quality candidate enzymes for genetic engineering technologies, such as microorganisms obtained in extreme environments, and optimizing the production and purification conditions of these enzymes to meet the needs of industrial applications. The application of biomics technology and advanced chemical analysis may also serve as effective tools, with the study of the synergistic mechanism of multiple enzymes possibly promoting the development of related fields. Therefore, to realize the production of value-added lignin compounds, we need to consider how to strengthen all aspects of lignin depolymerization, metabolism and product synthesis.

## Data Availability

All data generated or analyzed during this study are included in this published article.
